# A review of plant leaf disease identification by deep learning algorithms

**DOI:** 10.3389/fpls.2025.1637241

**Published:** 2025-08-20

**Authors:** Junmin Zhao, Laixiang Xu, Zizhen Ma, Juncai Li, Xiaowei Wang, Yunchang Liu, Xiaojie Du

**Affiliations:** ^1^ School of Computer and Data Science, Research Center of Smart City and Big Data Engineering of Henan Province, Henan University of Urban Construction, Pingdingshan, China; ^2^ School of Computer and Data Science, Research Center of Smart City and Big Data Engineering of Henan Province, Innovation Laboratory of Smart Transportation and Big Data Development of Henan Province, Henan University of Urban Construction, Pingdingshan, China; ^3^ School of Computer and Data Science, Henan University of Urban Construction, Pingdingshan, China

**Keywords:** plant disease control, plant leaf disease, deep learning, disease identification, convolutional neural network

## Abstract

Plant leaf disease control is crucial given the prevalence of plant leaf diseases around the world. The most crucial aspect of controlling plant leaf diseases is appropriately identifying them. Deep learning-based plant leaf disease recognition is a viable alternative to artificial methods that are useless and inaccurate. The proposed work aims to combine plant leaf disease datasets from various countries, review current research and progress in deep learning algorithms for plant disease recognition, and explain how different types of data are developed and used in this area using different deep learning networks. The feasibility of several network models for deep learning-based plant leaf disease detection is discussed. Solving shortcomings such as sunlight irradiation in plant planting conditions, similar disease incidence of different plant leaf diseases, and varied symptoms of the same disease in different damage periods or infection degrees are all essential study topics in the growth of this discipline. To address the concerns raised above and establish the field’s future development potential, we must research high-performance neural networks based on the benefits and downsides of diverse networks. The proposed work can serve as a foundation for future research and breakthroughs in the identification of plant leaf diseases.

## 1 Introduction

The ongoing strengthening of the greenhouse effect raises the Earth’s temperature and alters precipitation patterns. These factors significantly influence the occurrence, distribution, and transmission of plant leaf diseases. Pathogens and pests reproduce more quickly when temperatures rise (Chinnu et al., 2024). It leads to the rapid spread of pests and illnesses. Changes in precipitation patterns can lead to root damage or drought, lowering plant disease resistance. Plants are essential for managing the Earth’s climate. Plant diseases can worsen climate change, reduce crop quality and output, cause food shortages, and ultimately jeopardize human existence. Plant diseases can cause direct harm to plant tissues in a variety of ways, compromising plant aesthetics and market value. When it is severe, it can kill a huge number of plants. Early artificial identification of plant diseases had numerous limitations. Farmers have limited expertise. They have difficulties assessing the severity of disease progress. This would result in ineffective governance and excessive pesticide spraying, which will squander resources and pollute the environment (Strandberg et al., 2024). As a result, promptly, conveniently, and simply recognizing plant leaf diseases and improving plant disease control are critical for ensuring crop quality and yield, solving food concerns, sustaining human living conditions, and safeguarding the environment. This is also a significant motivation for studying this subject.

According to relevant information, there are many kinds of plant diseases. Plant diseases cause direct damage to plant tissues through parasitism, absorption of nutrients, destruction of cell structure, such as leaf spots, rot, fruit deformation, fruit cracking, etc. These damages not only affect the beauty of plants but also may reduce their market value; more serious ones can cause a large number of plant deaths (Roeswitawati et al., 2024). Accurate detection of plant leaf diseases is an important step in plant disease management. Due to the limitation of early technology, the identification of plant diseases is mainly manual identification, but due to the diversity and similarity of diseases and the low level of knowledge of some farmers, they cannot correctly judge the development degree of plant diseases, resulting in failure to prescribe the right medicine, thus causing the treatment process to be difficult to effectively control the development of plant diseases. In addition, in the process of treatment, a large number of pesticides are sprayed, which not only causes waste of resources but also causes environmental pollution and other problems (Abaineh et al., 2024). As a result, speedy, convenient, and simple detection of plant leaf diseases is critical to plant leaf disease control, as well as the field’s future development direction (Kalimuthu et al., 2021).

Although deep learning has achieved some results in identifying plant leaf diseases, it still faces numerous obstacles. How to improve the model’s generalization capabilities in multiple contexts and illness kinds while also better adapting to practical application scenarios. In the detection of plant leaf diseases, deep learning suffers from imbalanced data and erroneous labeling. This will reduce the model’s reliability and stability. Deep learning models that balance accuracy and computational resource consumption will be able to recognize plant diseases more efficiently on resource-constrained devices.

The proposed work discusses plant leaf disease recognition strategies based on on deep learning algorithms. Although deep learning did not attract widespread attention at the time due to hardware constraints, its potential gradually emerged. Krizhevsky et al. (2012) successfully implemented the famous convolutional neural network AlexNet on a graphics processor. At the Stanford ImageNet Large-Scale Visual Recognition Challenge, AlexNet made a breakthrough in classification accuracy far beyond other early computer vision methods. This accomplishment not only demonstrates deep learning’s formidable potential but also encourages the growth of diverse convolutional neural networks (CNN), quickening the field’s progress. Deep learning is being utilized more and more in the field of plant leaf disease identification, and its significance is becoming more and more apparent.

Deep learning is a branch of machine learning (Ganaie et al., 2022) that is based on artificial neural networks and learns complex data features through multiple hidden layers. Deep learning is the core technology to realize artificial intelligence, especially in the fields of image processing, speech recognition, natural language processing, and other significant breakthroughs (Matteo and J. Brendan, 2024). Deep learning models can automatically extract useful features from the original dataset, greatly reducing the burden of artificial feature recognition. And a deep learning model trained on a huge number of data points can learn the inherent law of data, even if new data does not show high prediction ability. Deep learning models may learn high-level and abstract data features, providing powerful representation capabilities (Rose and Rui, 2024). The subject of plant disease recognition has grown significantly as a result of deep learning intervention, with a number of reliable research outcomes emerging. Plant disease detection based on deep learning models has also become a hot spot in this field. Our work reviews the research and progress in plant leaf disease recognition based on deep learning models, as well as the challenges and opportunities faced by the field in recent years.

The suggested study examines the research and development of deep learning in plant leaf disease recognition. We present a thorough assessment of deep learning applications in plant disease recognition, taking into account recent research achievements. At the same time, we did a thorough review of the obstacles encountered in the field of plant disease diagnosis and investigated potential solutions. This establishes a clear path and reference for our future study. By summarizing and analyzing existing research findings, it is possible to encourage the further development of plant leaf disease identification technology, provide assistance in solving practical problems in plant disease control, and steer the field toward a more efficient, accurate, and practical direction.

We investigated convolutional neural networks, YOLO, structural optimization, and performance improvement strategies for lightweight models in disease recognition applications. We investigated the practical efficiency of several algorithms in difficult situations. It addresses accuracy, real-time performance, and the simultaneous detection of various diseases.

We mainly used Web of Science, the institute of electrical and electronics engineers (IEEE) Xplore, ScienceDirect, SpringerLink, and Google Scholar academic databases, focusing on literature from 2018 to 2025. This literature systematically reviews and integrates the research progress of deep learning in plant leaf disease recognition. The keywords we use are plant leaf disease, crop disease, deep learning, convolutional neural network, object detection, recognition, detection, and classification. Our inclusion criteria include deep learning-based leaf disease detection research, academic papers that use public or self-built field datasets, non-deep learning methods, pure theoretical models without image data, and research on non-leaf diseases such as root diseases. We acquired 580 papers after initial title and abstract screening and retained 210 following full-text reading. We also augmented 35 relevant articles with reference tracing. We supplemented and included over 20 prominent open-source datasets by searching Kaggle, GitHub, and academic institution official websites at the same time. We ultimately reviewed and integrated over 140 pieces of literature on algorithm creation, lightweight models, and multi-scenario applications.

The rest of the article is structured as follows: Section 2 offers a thorough analysis of each dataset and a thorough introduction to open-source databases both domestically and abroad. Plant species, sample size, and disease kinds are all included. Section 3 examines the specific performance and impacts of various implementation frameworks and provides an overview of plant leaf disease recognition algorithms based on one-stage detectors, two-stage detectors, and anchor-free frameworks. Section 4 examines the performance of alternative models and lightweight models under deep learning techniques. Section 5 concludes with a thorough analysis and forecast.

## 2 Plant leaf disease image database

Deep learning models, based on large data sets, can learn the inherent laws of data at a deeper level and have a higher accuracy rate for predicting new data that has not yet been seen (Mingle et al., 2024). Thus, datasets are the basis for building deep models, and more quantitative and high-quality datasets tend to build more successful deep learning models (Alsaghir et al., 2022). A high-quality deep learning model can more accurately and effectively identify the type of plant disease, disease degree, and other information. Relevant individuals can provide the appropriate medicine based on the results of the deep learning model, resulting in more scientific and effective governance outcomes. **Table 1** contains quality plant disease picture databases.

**Table 1:** Publicly available plant disease image data sets and websites.

Dataset	No. of Images	Disease Annotation	Source Organization	Datasets Links
PlantVillage	54,036	Expert	Penn State University	https://plantvillage.psu.edu/
CVPR 2020-FGVG7	3651	Expert	Plant Pathology Challenge competition	https://www.kaggle.com/c/plant-pathology-2020-fgvc7/data
Cucumber Plant Diseases Dataset	695	Expert	Hefei Institute of Intelligent Machinery, Chinese Academy of Sciences	https://www.kaggle.com/kareem3egm/cucumber-plant-diseases-dataset
New Plant Disease Dataset	87000	Expert	Colorado State University	https://www.kaggle.com/vipoooool/new-plant-diseases-dataset
PlantDoc	2598	Expert	Indian Institute of Technology	https://github.com/pratikkayal/PlantDoc-Dataset
Rice Diseases Image Dataset	5447	Expert	jonathando.dev	https://www.kaggle.com/minhhuy2810/rice-diseases-image-dataset
PlantPathology Apple Dataset	3171	Expert	Plant Pathology Challenge competition	https://www.kaggle.com/lextoumbourou/plantvillageapplecolor
New Plant Diseases Dataset (Augmented)	22900	Expert	Penn State University	https://www.kaggle.com/noulam/tomato
PlantifyDr Dataset	125000	Expert	Indian Institute of Technology	https://www.kaggle.com/lavaman151/plantifydr-dataset
Corn Leaf Diseases(NLB)	4115	Expert	Kathmandu University	https://www.kaggle.com/rabbityashow/corn-leaf-diseasesnlb
Durian Leaf Disease Dataset	4437	Expert	Ton Duc Thang University	https://www.kaggle.com/datasets/cthng123/durian-leaf-disease-dataset
Banana Leaf Spot Diseases Dataset	5909	Expert	Bangabandhu Sheikh Mujibur Rahman Agricultural University	https://www.kaggle.com/datasets/shifatearman/bananalsd
Jute Leaf Disease Dataset	1820	Expert	Varendra University	https://www.kaggle.com/datasets/srkuhin/jute-leaf-disease-detection
Potato Disease Leaf Dataset (PLD)	4062	Expert	eFAIDA TECHNOLOGIES, University of Okara	https://www.kaggle.com/datasets/rizwan123456789/potato-disease-leaf-datasetpld
Potato Leaf (Healthy and Late Blight)	426	Expert	Software Engineer at Velou	https://www.kaggle.com/datasets/nirmalsankalana/potato-leaf-healthy-and-late-blight
Eggplant Disease Recognition Dataset	3136	Expert	Marketing Manager at Jazira & Victoria Ltd	https://www.kaggle.com/datasets/kamalmoha/eggplant-disease-recognition-dataset
Strawberry Disease Detection Dataset	2500	Expert	Jeonbuk National University	https://www.kaggle.com/datasets/usmanafzaal/strawberry-disease-detection-dataset?select=train
PDD271	220592	Expert	Beijing Puhui Sannong Technology Co. Ltd	https://drive.google.com/file/d/1QMR1bUfEuMbZz-Mb3u2IXdbMgz7oj2Pe/view
Agricultural Disease and Pest	100000	Expert	Hefei Institute of Physical Sciences, Chinese Academy of Sciences	http://www.icgroupcas.cn/website_bchtk/index.html
Rice Leaf Diseases Data SetCotton plant	120	Expert	Dharmsinh Desai University	https://archive.ics.uci.edu/ml/datasets/Rice+Leaf+Diseases
Cotton plant diseases	234	Expert	Personal	https://www.datacastle.cn/dataset_description.html?type=dataset&id=2465
Plant disease recognition dataset	1530	Expert	University of Asia Pacific	https://www.kaggle.com/rashikrahmanpritom/plant-disease-recognition-dataset
Sugarcane Plant Diseases Dataset	19926	Expert	University of Washington	https://www.kaggle.com/datasets/akilesh253/sugarcane-plant-diseases-dataset
soybean.leaf.dataset	6410	Expert	Universidade do Estado de Mato Grosso	https://www.kaggle.com/datasets/maeloisamignoni/soybeanleafdataset
PDDB	50000	Expert	Embrapa CNPTIA	https://www.digipathos-rep.cnptia.embrapa.br/jspui/

### 2.1 Foreign major plant leaf disease image database

1. Plant village. This dataset is a public dataset created jointly by David Hughes and Marcel Salathé and is one of the most used databases today. Plant Village includes 14 plants and 26 diseases, a total of 38 categories, including 54,036 images, which is very suitable for plant disease detection and recognition model training, but most of the images in Plant Village are taken in the laboratory or under a single background, and fewer images are taken under complex natural conditions.
2. Plant pathology 2020-FGVC7. Mainly high-quality annotated apple images, including apple scab, apple rust, multiple disease coexistence, and healthy leaves, totaling 3651 images. Among them, there are 1200 images of apple scab, 1399 images of apple rust, 187 images of coexistence of multiple diseases, and 865 images of healthy leaves.
3. Cucumber plant diseases dataset. The dataset, shared by Karim Negm, contains 695 images of diseased and healthy cucumbers taken under natural conditions in the field.
4. New plant disease dataset. The data set was recreated by Samir Bhattarai using data enhancement techniques. The dataset consists of 38 different categories, including 87,000 healthy and non-healthy leaves, but the image background of the data is a single background.
5. PlantDoc. Created by Singh et al (Singh et al., 2020), it contains 2598 images covering 13 plant species and 17 disease types.
6. Rice diseases image dataset. Covering brown spot disease, leaf spot disease, ironworm disease, and healthy leaves, a total of 5447 images.
7. Plant pathology apple dataset. Derived from Plant-village, including apple scab, black rot, cedar apple rust, and healthy leaves, containing 3171 images.
8. New plant diseases dataset (Augmented). The dataset is a dataset of related tomatoes, derived from PlantVillage data through data augmentation techniques, including 9 diseased tomatoes and 1 healthy leaf, for a total of 22,900 images.
9. PlantifyDr dataset. The dataset contains 10 different plant types, primarily apples, bell peppers, cherries, oranges, corn, grapes, peaches, potatoes, strawberries, and tomatoes. 125,000 images of 37 plant disease types.
10. Plant disease recognition dataset. The dataset contains 1530 images of healthy, powdery, and rusty.
11. Corn leaf diseases (NLB). 4115, including diseased and healthy corn.
12. Durian leaf disease dataset. The dataset includes images of different types of durian leaf disease, including algal blotch, heteroscab, leaf blight, and phomopsis leaf blotch, and images of healthy leaves. There are 4437 images.
13. Banana leaf spot diseases dataset. The dataset covers the three major banana leaf spot diseases, cigatoka, kodana, and polychaete, as well as images of healthy banana leaves. The dataset consists primarily of two subsets. The original set contains 937 images, which are divided into 4 categories and provided in JPG format. They are the most original image data and visually show the various states of banana leaves. The enhanced set is extended by a series of advanced enhancement techniques on the basis of the original set. These enhancement techniques include Gaussian blur, horizontal flip, cropping, linear contrast adjustment, cropping, translation, rotational cropping, etc. Through these operations, 400 images are added to each class, making the total number of images in the enhancement set reach 1600.
14. Jute leaf disease dataset. There were 609 images of cercospora leaf spot in the comprehensive data set of jute crop disease detection. There are 647 images of golden mosaic in the dataset.
15. Potato disease leaf dataset (PLD). The Potato Leaf Disease dataset contains 4062 images collected from the Punjab region of central Pakistan.
16. Potato leaf (Healthy and Late Blight). Heterogeneous image datasets were collected from potato farms in Holeta, Ethiopia, with the help of two plant pathologists. The dataset is correctly labeled with two classes, “Healthy” and “Late Blight,” and the images are diverse, meaning some images were captured using less noisy background images while others were captured using highly noisy environments. 63 images were collected under the category of “late blight,” and 363 images were collected under the category of “health.” Finally, the data sets prepared can be used for different studies aimed at plant disease detection and classification.
17. Eggplant disease recognition dataset. This dataset includes seven different eggplant diseases: healthy leaves, pest disease, leaf spot disease, mosaic virus disease, small leaf disease, white mold, and blight. The dataset was based on the original 392 eggplant images, and various technical image processing techniques were applied by increasing the number of data points, resulting in a total of 3136 enhanced photos from the original images.
18. Strawberry disease detection dataset. It includes 2500 images and provides segmentation annotation files for 7 different types of strawberry diseases.
19. Sugarcane plant diseases dataset. The dataset contains 19926 images of sugarcane leaves. Among them, 4800 were bacterial blight, 3132 healthy leaves, 2772 mosaic leaves, 3108 red rot, 3048 rust, and 3030 yellow rot.
20. soybean.leaf.dataset. The dataset consists of three image folders: Caterpillar, Diabrotica Speciosa, and Healthy, for a total of 6410 images. Caterpillar 3309 images, Diabrotica Speciosa 2205 images, and healthy 896 images.

### 2.2 Plant leaf disease image database in China

1. PDD27. The plant disease dataset PDD271 collected by Liu et al. (2021) includes 220,592 plant leaf images covering 271 plant disease categories. Each plant disease category contains at least 500 images of more than 200 plants. And each image is marked by experts in time when it is collected. After collection, experts who are not involved in the labeling work check to ensure the correctness of the label.
2. Agricultural disease and pest research database (IDADP). The dataset is a comprehensive database jointly constructed by the Hefei Institute of Intelligent Machinery, the Institute of Subtropical Agroecology, and the Institute of Remote Sensing and Digital Earth, China Academy of Sciences, covering various plant types such as field crops, fruits and vegetables, and various disease types such as fungi, bacteria, and viruses. Each pest includes hundreds to thousands of images. Most of the images were taken with SLR cameras with a resolution of no less than 20 million pixels (6000×4000, 5472×3648), and a few were taken with mobile phones with a resolution of 4128×2322 pixels. Moreover, most images are taken under natural conditions and can be applied to the identification and detection of plant diseases under complex natural conditions.
3. Plant disease symptom database (PDDB). The dataset is a free database covering 21 plant species, 171 disease types, and nearly 50,000 images collected. Eighty-five percent of the images were taken under realistic conditions, and the rest were taken under controlled conditions. The images were all captured by digital cameras and mobile devices with resolutions ranging from 1 to 24 megapixels (Barbedo et al., 2018) and were annotated by experts.
4. Rice leaf disease dataset. The dataset images were taken under direct sunlight against a white background, mainly for rice bacterial blight, brown spot, and smut, with 40 shots of each disease, totaling 120 shots.
5. Cotton plant disease. The dataset contains 1522 high-quality images of cotton pests and diseases.

## 3 Advances in plant leaf disease detection

Plant leaf disease detection is the process of recognizing and locating infected areas, as well as the precise location of plant leaf disease in a complicated natural environment (Abade et al., 2021). This technology is the basis for realizing accurate classification and identification of plant leaf diseases and evaluating the damage degree of diseases. It is also the key link for accurately locating disease areas and guiding plant protection equipment to implement precise spraying.

Early plant disease target detection algorithms usually use a sliding window strategy to generate candidate regions, then extract the features of these regions and classify them with classifiers to finally determine the target regions (Ben et al., 2024). Viola-Jones detection, histogram of oriented gradient detection, and deformable component modeling are examples of common techniques. The sliding window method traverses the image by setting different scales and aspect ratios. Although this method can ensure that no potential disease regions are missed, it will produce a large number of redundant candidate windows, resulting in a significant increase in computational effort. In addition, it takes a lot of time to traverse the whole image, which makes the detection efficiency low. On the other hand, the feature extraction of candidate regions depends on manual design, and the extracted features mainly focus on the underlying information, such as the color and shape of diseases, whichamakes the robustness of disease detection poor. The a daptive boosting (AdaBoost) and support vector machine (SVM) are usually used in classifiers, but these methods have the problems of slow recognition speed and low accuracy.

### 3.1 Plant leaf disease detection based on target detection framework

Emerging detection algorithms such as the deep learning-driven region-based convolutional neural networks (R-CNN) series, you only look once (YOLO), single shot multi-box detector (SSD), and CenterNet show significant performance advantages over earlier plant target detection algorithms. In the object detection framework of deep learning, these algorithms can be divided into two main categories: two-stage detectors and one-stage detectors (Bsher et al., 2024).

#### 3.1.1 Plant leaf disease detection based on two-stage detectors

**Figure 1:**
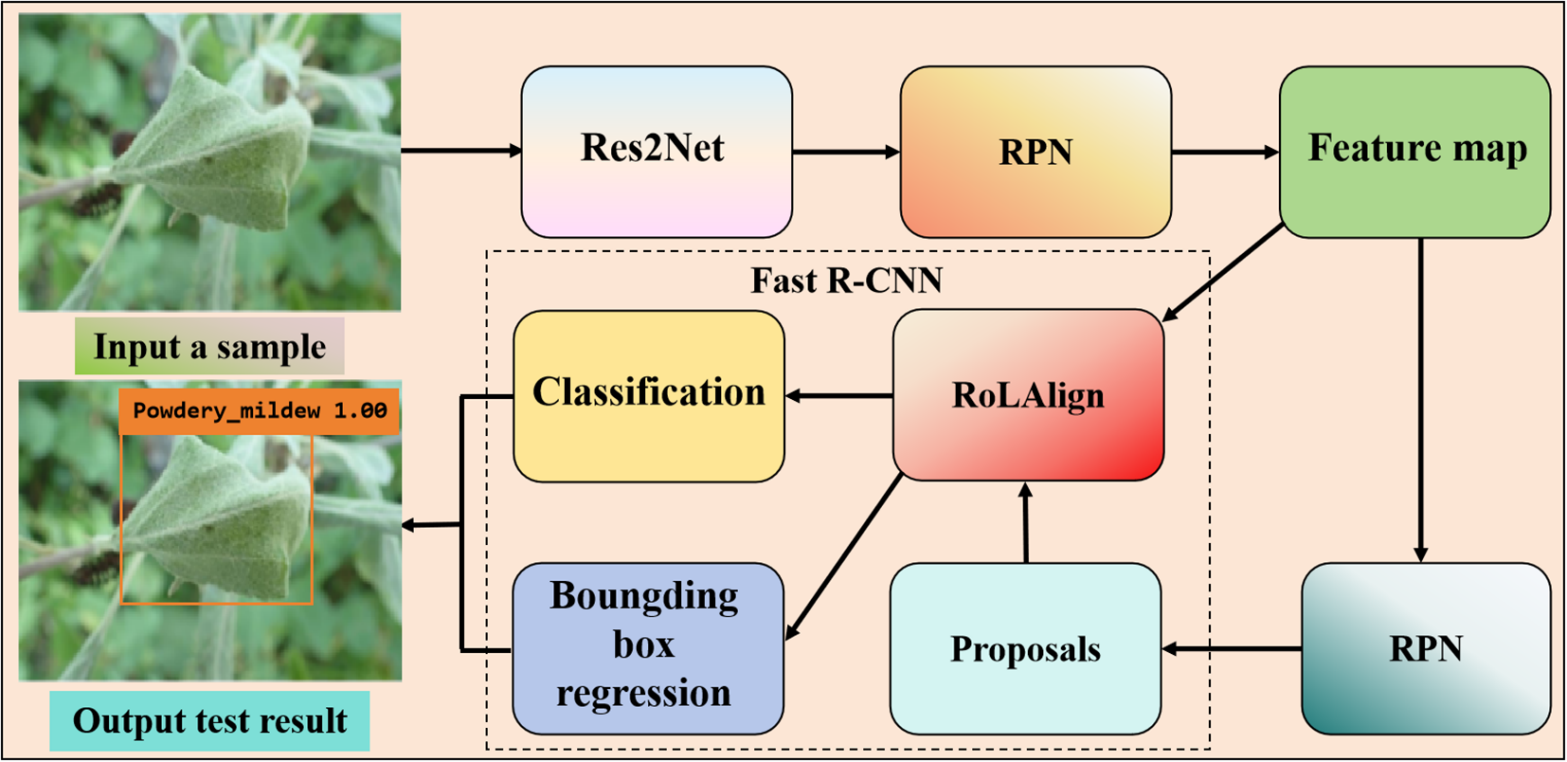
Two-stage detection algorithm based on apple powdery mildew leaf diseases. The flowchart of the detection model using Faster R-CNN. The result picture contains the predicted disease category, the confidence, and the location of the disease marked by an orange box. The region proposal network (RPN) generates multiple anchor boxes with preset proportions and scales on the feature map, and then performs classification and coordinate regression to screen out candidate boxes that may contain the target.

Two-stage first generates a sparse set of candidate boxes using a candidate box generator, extracts features from each candidate box, and then predicts the category of the candidate box region using a region classifier in **Figure 1**.

Li M. et al. (2023) designed an integration model integrating one-stage and two-stage target detection networks. The YOLO and Faster-RCNN are integrated to maximize the size of the target frame using a clustering approach, hence improving the target’s detection effectiveness. Transfer learning was used to improve the training speed of the model, and finally the average accuracy of 37 pests and 8 diseases was 85.2%, which was significantly higher than other control models. Gong and Zhang (2023) suggested an enhanced Faster R-CNN approach for detecting apple leaf disease. Res2Net and feature pyramid network are described as feature extraction networks. A region of interest align (RoIAlign) replaced region of interest pooling (RoIPool) to increase candidate region accuracy. The accuracy rate is 63.1%. Wu and Jiang (2023) created a pine wilt disease (PWD) detection and extraction algorithm based on Mask R-CNN. Firstly, the advanced ConvNeXt network is used to improve image feature extraction. In order to improve data sharing under low batch-size training, the original multi-scale structure is converted to PA-FPN and normalized. Lastly, a branch is introduced to the Mask module to enhance the fusion-based object extraction capacity. The improved method achieves 91.9% on the PWD dataset. Zhao S. et al. (2022) reported a new Faster R-CNN architecture, composed of multi-scale fusion ResNet, FPN, and convolutional block attention module (CBAM) blocks, which can effectively extract rich strawberry disease features. Compared with Mask R-CNN and YOLO-v3, this model has higher accuracy and faster detection operation requirements, and its accuracy can reach 92.18%. Wang M. et al. (2023) exhibited an on-site sweet potato leaf detection method based on a modified Faster R-CNN framework and visual attention mechanism. The accuracy of the investigated method reaches 95.7%, 2.9% higher than the original Faster R-CNN and 7% higher than YOLOv5. The constructed method achieves good performance in detecting dense leaves or occluding leaves. Li et al. (2022) used a convolutional neural network for multi-scale feature fusion in their potato leaf disease detection approach. The approach offered a workable solution for the diagnosis of maize leaf disease because its average accuracy is higher than that of YOLOv5, Faster R-CNN, and CenterNet.

A convolutional neural network (CNN), based on regional recommendations, has achieved some results in the field of plant leaf disease detection, displaying innovation and application potential, although there are still numerous hurdles. From a data standpoint, several studies use synthetic data to improve model performance. Although this method increases accuracy, there are distinctions between synthesized and real-world data. This may have an impact on the model’s capacity to generalize in complex real-world scenarios. In terms of model complexity, some models include numerous sophisticated network structures. Although this method improves feature and object extraction capabilities, it also results in higher processing costs and lengthier training and inference periods. This presents certain issues for the deployment of limited resources in agricultural areas. When it comes to model comparison, different research employs different datasets and evaluation metrics, making it impossible to compare the benefits and drawbacks of models objectively. Some research concentrates on various plant diseases, but their indicators lack a common reference. This is not favorable to technical discussions. Additionally, the model’s interpretability is often poor. Deep learning, as a black box, makes it difficult for farmers to understand its decision-making basis, reducing confidence and acceptability in practical implementations.

**Figure 2:**
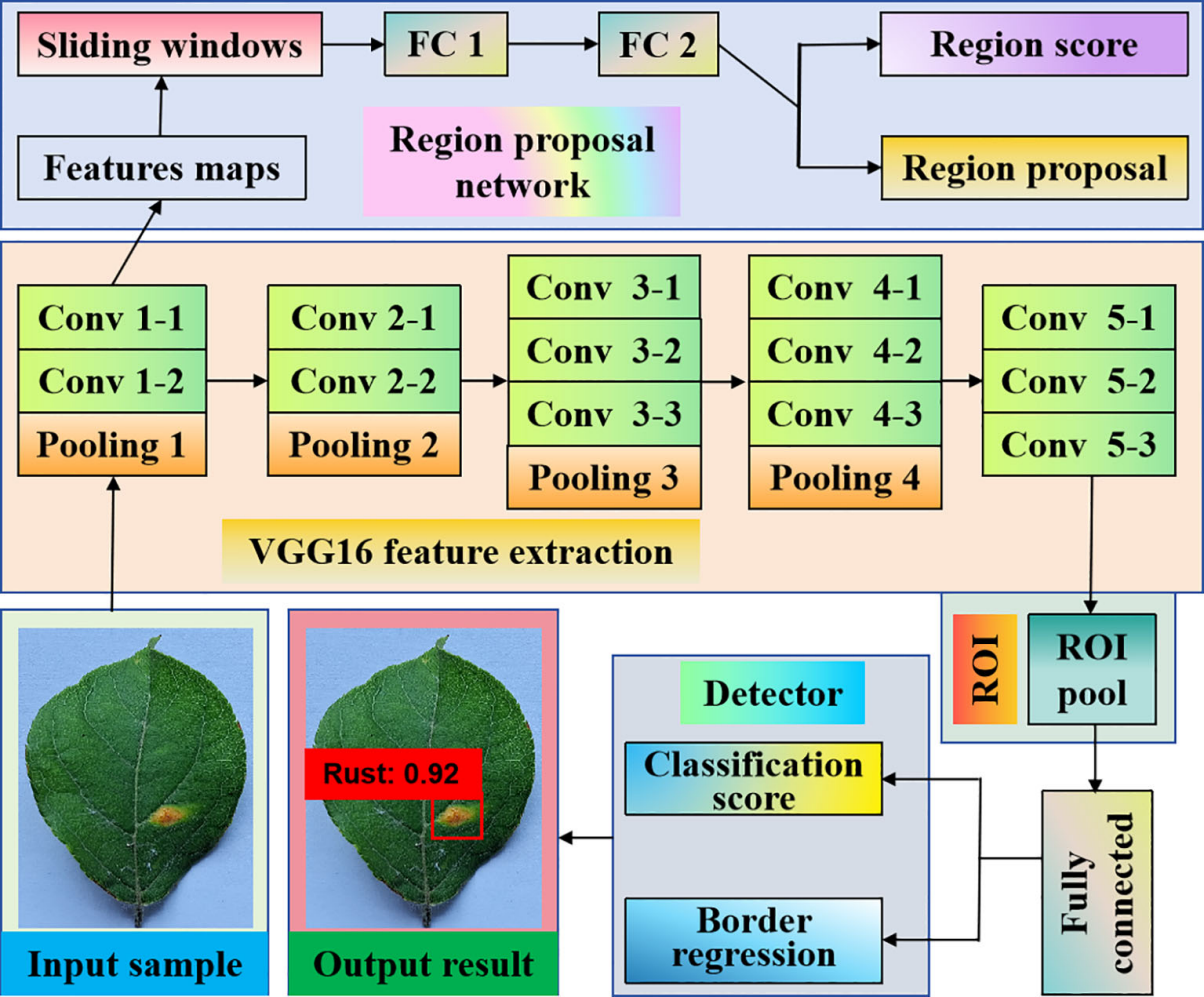
Two-stage detection algorithm based on apple rust leaf diseases. It achieves high-precision object detection performance through a two-stage network and a regional proposal network. Compared with other one-stage networks, Faster R-CNN is more accurate in handling multi-scale and small target shortcomings.

There are R-CNN, Fast-RCNN, and Faster-RCNN. Haruna et al. (2023) developed a data augmentation pipeline that used style-generative adversarial network adaptive discriminator augmentation and laplacian filter variance to improve Faster-RCNN performance in detecting major rice leaf diseases. Compared with the standard data, this method achieves an average accuracy of 93% in rice leaf disease detection, and the Faster R-CNN model has a significant improvement in rice leaf disease detection. The structure of Faster R-CNN is manifested in **Figure 2**.

#### 3.1.2 Plant leaf disease detection based on one-stage detector

**Figure 3:**
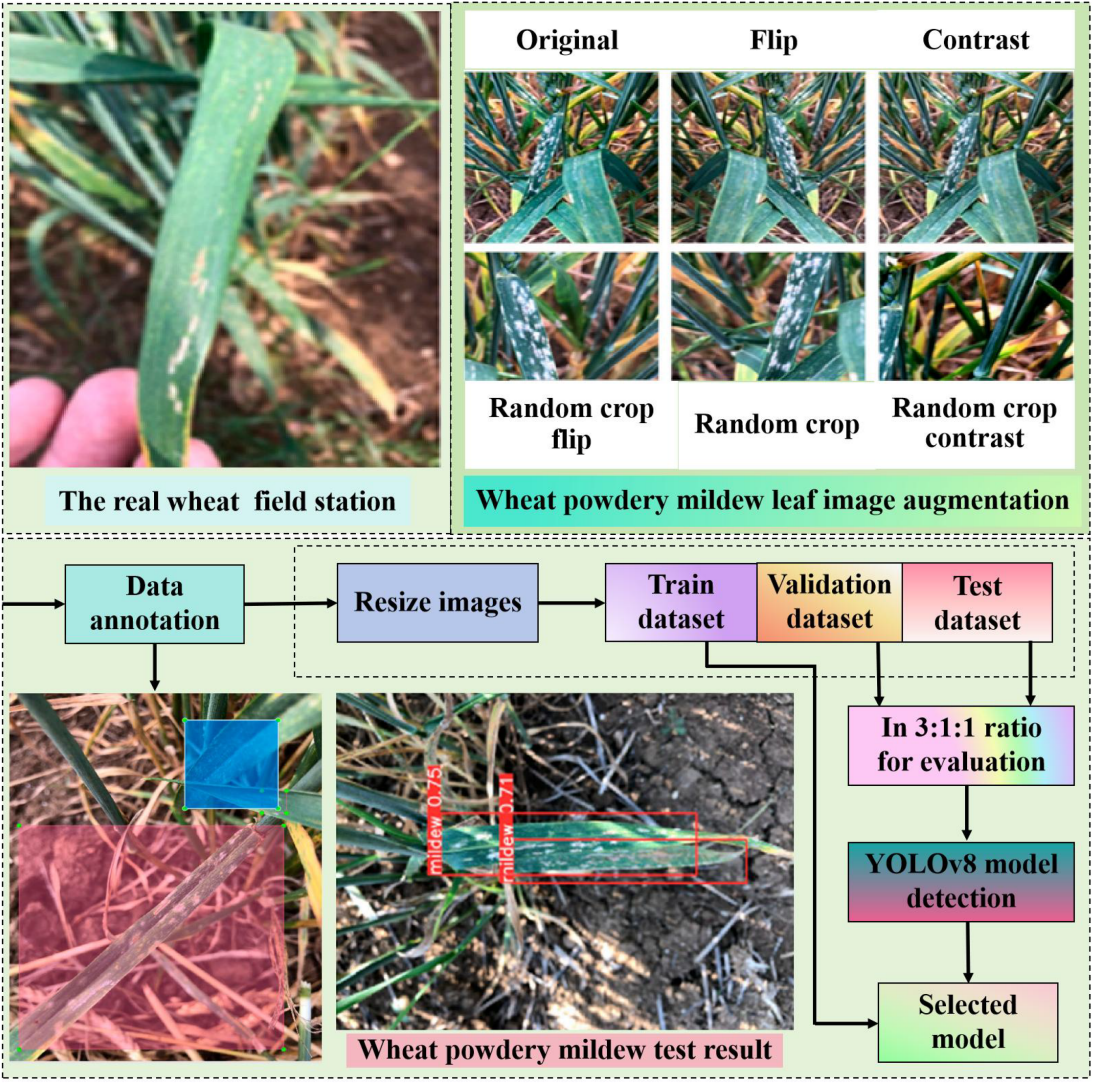
One-stage detection algorithm diagram for wheat powdery mildew. Wheat powdery mildew is detected using YOLOv8. There are 896 photos in this dataset. Its average accuracy, recall, and F1 score are 0.79, 0.74, 0.770, 0.76, and 0.35, respectively.

One-stage detectors make category predictions directly for objects at each position on the feature map, without going through the region suggestion step in two-stage detectors (Önler and Köycü, 2024). As shown in **Figure 3**.

**Figure 4:**
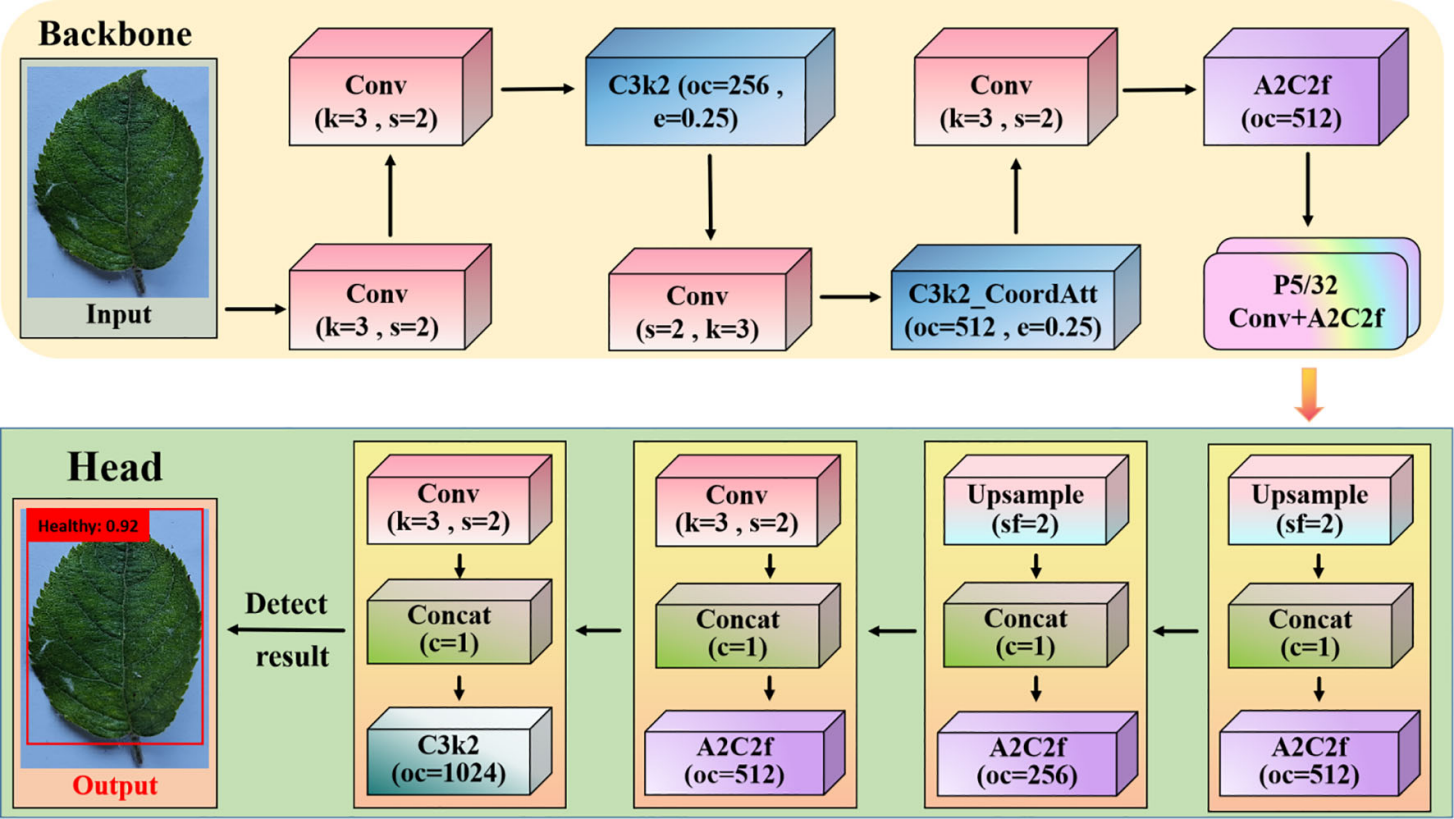
Apple leaf disease detection for YOLOv12. k is the height and width of the filter in the convolution operation, s is the step size that slides on the input feature map during convolution or upsampling, oc is the channel dimension of the output feature map of the convolution/module, c is the feature fusion operation performed on the c-th dimension, and sf is the scaling factor used in the upsampling module.

There is YOLO, SSD, and their variants. YOLO employs a single neural network to simultaneously predict bounding boxes and probabilistic images of multiple objects within a range. YOLO, unlike existing approaches, employs a real-time detection algorithm that divides the input image into grids and predicts the cell contents of bounding boxes based on those grids. Real-time performance sacrifices accuracy for fine-grained localization but remains competitive in object detection, making it suitable for a wide range of applications. YOLOv12, one of the newest versions of object-detection architectures, has shown great promise in situations requiring both high detection accuracy and real-time inference. The outstanding YOLOv12 is presented in **Figure 4**.

Devisurya et al. (2022) used YOLOv3 Tiny to detect turmeric illnesses (leaf spot and root rot disease). This approach outperforms existing YOLO variations, including the Faster R-CNN based on the visual geometry group (VGG16). Arun et al. (2024) employed T-YOLO v4 to detect rice leaf disease. This approach builds on YOLOv4 by including a YOLO detection layer, spatial pyramid pooling, a convolutional block attention module, an hourglass feature extraction module, and a Ghost module. It outperformed the YOLOv7 model in terms of recognition accuracy, scoring 86.36%. Daniela et al. (2024) used YOLOv7, YOLOv8, and YOLO-NAS to annotate the entire leaf disease area and achieved an accuracy of 97.9%. However, in the task of whole pod disease detection, YOLOv7 and YOLOv8 performed better, with accuracy rates exceeding 95%. Liu et al. (2016) designed a multi-scale prior box for YOLO to address defects with low accuracy and inaccurate localization in small object detection. Tian et al. (2022) proposed an improved SSD method for detecting apple leaf diseases. This method achieved an accuracy of 83.19% and a detection speed of 27.53 FPS on the apple leaf disease test set.

Guo et al. (2022) offered a cotton detection method based on improved SSD. This method introduces the lightweight network MobileNetV2 to improve the backbone feature extraction network and then integrates the SE attention mechanism, the ECA attention mechanism, and the CBAM attention mechanism. The improved SSD_MobileNetV2+ efficient channel attention (ECA) model has higher accuracy than the SSD_VGG network model in complex cases, reaching 84.8%. Yin et al. (2024) applied a disease point identification method based on a SSD network, which improved the accuracy of disease point identification. The learned parameter information is transferred to the backbone feature extraction network of the SSD model, which shortens the blob detection time to 0.14 s. When the intersection over union (IoU) threshold is 0.5, the mean average precision at IoU=0.5 (mAP@0.5), the average accuracy of the algorithm reaches 97.1%, which is about 6.35% higher than that of the original algorithm. Compared to existing algorithms such as YOLOv5 and Faster R-CNN, mAP0.5 improves by 16.84% and 8.61%, respectively. Although improved and optimized continuously, the one-stage detection algorithm has been improved in both accuracy and speed. mAP@0.5 is the primary metric for determining the model’s localization ability in object detection tasks. It assesses the model’s approximation detection performance on the target by applying a 50% IoU threshold. It is appropriate for screening models that meet fundamental positioning requirements (e.g., existence judgment or counting tasks), and prioritizing models with high indicators (e.g., the YOLO series) allows for a balance of efficiency and accuracy. However, it is unaffected by small targets, thick sceneries, or fine border detection and is easily influenced by simple sample distributions.

From the standpoint of algorithm universality, several researchers optimize algorithms for specific plant diseases. Although they had good findings, the model’s universality was not optimal. When confronted with different types of plants, illnesses, and complicated and diverse actual agricultural production conditions, the performance of these models may suffer. A large amount of data collecting and model training effort must be redone.

In terms of precision and speed, while YOLO achieves real-time performance by foregoing some fine-grained positioning, this balance is not ideal in practical applications. As the model becomes more complicated, it may have an impact on real-time detection performance. How to improve detection speed and establish a more perfect balance while maintaining high accuracy is an important subject that must be addressed in future study.

Although SSD solves YOLO’s problem of inaccurate small object detection and localization, it also has its own limitations. It faces more complex and varied actual disease scenarios, and its robustness still needs further verification. There is a lack of unified standards and comprehensive evaluation for comparing the effects of different improvement methods, making it difficult to determine which improvement strategy is more universal and superior.

In addition, current research mostly focuses on improving and optimizing the algorithms themselves, with relatively less attention paid to data quality and annotation. High-quality and diverse data are the foundation for training high-performance models, but obtaining large-scale, high-quality, and accurately labeled plant disease data in actual agricultural production is not an easy task. The noise and annotation errors in the data may have a negative impact on the training and performance of the model. Effective data cleaning and annotation optimization methods face certain challenges.

### 3.2 Foreign major plant leaf disease image database

Zhou et al. (2019) introduced CenterNet, an anchor-free detection algorithm that is based on CornerNet and replaces the original detection of two key points (i.e., the upper left and lower right corners of the picture) with the estimation of the image’s center point. Because this technique avoids the need to generate an anchor box and estimate loss using a thermal map, it saves time and improves detection performance significantly. CenterNet-based illness detection is currently understudied. However, it has been shown to be relevant to target identification in natural conditions.

Marriam et al. (2023) provided the CoffeeNet model to detect various infections in coffee plant leaves. The model was compared against other deep learning models, including GoogLeNet, AlexNet, and EfficientNet-B0. The results revealed that CoffeeNet outperformed in terms of recognition accuracy and efficiency, with an accuracy rate of 98.54%, as well as processing time and disease-specific key point localization. Liu W. et al. (2023) designed an improved YOLOX tomato leaf disease identification method. They replace the YOLOX backbone network with MobileNetV3 for lightweight model feature extraction and add a CBAM module. According to simulation experiments and field tests, YOLOX’s accuracy rate increased by 1.27%, up to 98.56%. Albattan et al. (2021) constructed a CornerNet model based on DenseNet-77 to detect 10 types of tomato leaf diseases. By modifying CornerNet, feature extraction, and classification, the model can accurately locate disease regions. Compared with other object detection technologies, this model still shows high reliability under conditions such as noise, illumination changes, color changes, and size changes. The average accuracy value reaches 98.4%, which is higher than the average accuracy value of the comparison method. 12.42% higher.

Yi et al. (2024) created a model for identifying and locating citrus Huanglong disease. The accuracy rate of the upgraded model was 7.9% higher than that of the RT-DETR-r18 model, and it demonstrated notable progress in a number of important metrics. The highest accuracy of the model is 92.7%. The method ensures accurate location and identification of citrus Huanglong disease in complex and diverse environments. The detection algorithm without an anchor frame is superior to the detection algorithm based on an anchor frame in performance, and it will be the main research direction in disease area detection in the future.

The anchor-free box detection algorithm has become an important technology in the field of plant leaf disease detection due to its advantages of simplified process, improved efficiency, and strong robustness. Although current research is based on frameworks such as CenterNet and CornerNet, and high-precision detection is achieved through CoffeeNet and HHS-RT-DETR, the resolution of small targets is limited, and the deployment of edge devices is still difficult to overcome. Future research should focus on two directions: first, developing lightweight anchor-free models suitable for field edge devices using neural architecture search and model compression techniques, and second, incorporating mechanisms such as dynamic feature pyramids and high-resolution heatmaps to improve the sensitivity of detecting small lesions. Plant disease detection systems will advance toward real-time precision and universality as the anchor-free paradigm, transformer architecture, and multimodal perception technologies are fully integrated. This results in more effective technical support for smart agriculture.

### 3.3 Analysis and prospect of plant leaf disease detection

**Tables 2**–**4** present an analysis of the state of research on intelligent plant disease detection technology. The identification of diseases in soybeans, corn, potatoes, and other important commercial crops is the main emphasis of the current study. Good detection performance is demonstrated by both one-stage and two-stage detection models. Though pathological features without distinct edges are frequently left out of the detection range, there are notable variations in the criteria used to define lesion areas. For example, some studies independently identify large-area lesions on single leaves, while others use an overall identification strategy for densely distributed small lesions. It should be noted that there are still technical bottlenecks in the application of existing algorithms in actual farmland environments, especially in the face of high-density small target detection. It is necessary to enhance the current models’ adaptability under dynamic environmental settings such as complicated lighting, background interference, and target occlusion.

**Table 2:** Plant leaf disease detection based on one-stage detector.

Authors, Years	Class	Total	Collect ways	Methods	Performance	DOI
Di and Li (2022)	Apple	1404	Plant Village	Tiny-YOLO	Precision=0.938 Recall=0.99 Accuracy=0.99 IoU=0.805 mAP=0.9981	10.1371/jou- rnal.pone.0262629
Soeb et al. (2023)	Tea	4000	Camera	Improved YOLOv 7	Precision=0.967 Recall=0.964 F1-Score=0.965 Accuracy=0.973 Train Time =19430s mAP=0.982	10.1038/S41598-0 23-33270-4
Luo et al. (2023)	Citrus	3202	Online	Light-SA YOLOV 8	Precision=0.926 Recall=0.894 F1-Score=0.91 Model size=4.5MB	10.1109/ACC ESS.2023.3340148
Lin et al. (2023)	Tea	1000	Drone	TSBA-YOLO	Precision=0.8783 Recall=0.8527	10.3390/f 14030619
Zhang D. et al. (2024)	Wheat	20000	Microscope	GSD-YOLO	Precision=0.938 Recall=0.954	10.3390/agriculture14122278
Sun et al. (2024)	Apple	4863	Online	YOLOv 5-Res	Precision=0.814 Recall=0.769 Model size=10.8MB mAP=0.869	10.3390/agronomy14061331
Yuan et al. (2024)	Pine	11200	Drone	Light-ViTeYOLO	Recall=0.957	10.3390/f15061050
Li Y. et al. (2024)	Rubber	6200	Camera	PM-YOLO	Precision=0.848 Recall=0.856 F1-Score=0.852 mAP=0.869	10.3390/insects15120937
Zhang S. et al. (2024)	Jute	3252	Baidu Google	JutePest-YOLO	Precision=0.9872, Recall=0.949 F1-Score=0.9677	10.1109/ACCESS.2024.3403491
Ye et al. (2024b)	Tea	3743	Camera	Improved YOLOv 8	Precision=0.8747 Recall=0.8917 F1-Score=0.8831 mAP=0.9526	10.3390/plants13101377
Chen Z. et al. (2024)	Grape	10000	Camera	YOLOv8-ACCW	F1-Score=0.924 mAP=0.928	10.1109/ACCESS.2024.3453379
Meng et al. (2025)	Maize	14700	Camera	YOLO-MSM	Precision=0.9011 Recall=0.8264 TrainTime= 3312s	10.1038/s41598-025-88399-1
Zhu et al. (2025b)	Grape	17642	Plant Village	YOLOv8	Precision=0.9264 Recall=0.9328	10.1371/journal.pone.0321788
Liu Z. et al. (2025)	Tomato	2646	Plant Village	YOLO-BSMamba	Precision=0.858 Recall=0.784 F1-Score=0.819	10.3390/agronomy15040870

**Table 3:** Plant leaf disease detection based on two-stage detector.

Authors, Years	Class	Total	Collect ways	Methods	Performer	DOI
Liu H. et al. (2022)	Maize	7222	Field shot	LS-RCNN and CENet	Accuracy=0.99 Precision=0.99 recall=0.99 F1-score=0.30	10.1038/S41598-022-23484-3.
Nawaz et al. (2022)	Tomato	54306	PlantVillage	Faster-RCNN	Accuracy=0.98 Precision=0.99 recall=0.99 F1-score=0.99 mAP=0.98 IoU=0.93	10.1038/S41598-022-21498-5
Gong and Zhang (2023)	Apple	4182	Mobile phone	Faster R-CNN	Accuracy=0.63 Precision=0.63 recall=0.71 IoU=0.75	10.3390/agriculture 13020240.
Sun et al. (2023)	Tomato	5090	Camera	Veg DenseCap	Accuracy=0.88 Precision=0.99 mAP=0.93 IoU=0.70	10.3390/agronomy13071 700.
Zhou et al. (2024)	Rice	10409	Field	R-CNN and YOLOv	Accuracy=0.98 Precision=0.91 recall=0.95 F1-score=0.99 mAP=0.95 IoU=0.95	10.3390/AGRICULTURE14020290.
Zhang Z. et al. (2024)	Tea	1692	Camera	Faster RCNN	Accuracy=0.87 Precision=0.87 recall=0.90	10.1016/J.SCIENTA.2024.112949.
Liu J. et al. (2025)	Rice	3754	Camera	Faster R-CNN	Accuracy=0.83 mAP=0.83 IoU=0.50	10.5755/j01.itc.54.1.39520.
He J. et al. (2025)	Maize	7928	Plant Village	YOLOv11-RCDWD	Accuracy=0.92 Precision=0.92 recall=0.85 F1-score=0.88 mAP=0.66	10.3390/app15084535.
Yadav and Tewari (2025)	Tomato	2786	Fieldplant	CONF-RCNN	Accuracy=0.90	10.1007/s41348-024-01057-y.

**Table 4:** Plant leaf disease detection based on anchor-free.

Authors, Years	Class	Total	Collect ways	Methods	Performance	DOI
Bao et al. (2022)	Tea	700	Camera	AX-RetinaNet	Precision=0.9675 Recall=0.94 F1-Score=0.954 mAP=0.9383	10.1038/s41598-022- 06181-z
Li X. et al. (2023)	Multiple plant	58486	PlantVillage	VLDNet	Precision=0.983 Recall=0.9832 F1-Score=0.9831 Accuracy=0.9832	10.3390/agriculture13081482
Hu X. et al. (2023)	Strawberry	3411	Field	CALP-CNN	Precision=0.9255 Recall=0.918 F1-Score=0.9196 Accuracy=0.9256	10.3389/fpls.2023.1091600
Chen et al. (2023)	Oilseed rape	1764	Controlled	AMDFNet	Precision=0.8461 Recall=0.8495 Accuracy=0.8678	10.3390/plants12142701
Shao et al. (2024)	Cotton	3910	Field shot	CANnet	Precision=0.988 F1-Score=0.986 Accuracy=0.986	10.3390/agriculture14091577
Li S. et al. (2024)	Tea	658	Camera	VCRUNet	Precision=0.9227 Recall=0.9237 F1-Score=0.9232 Accuracy=0.9248	10.1109/ACCESS.202 4.33 73707
Liu and Wang (2024)	Multiple plant	11564	Controlled	MIFV	Precision=0.9353 Recall=0.9122 mAP=0.9238	10.1186/s12870-024-05346-4
Ye et al. (2024a)	Tea	4560	Camera	YOLOv8-RMDA	Precision=0.8484 Recall=0.8821	10.3390/s240928 96
Wang J. Y. et al. (2024)	Tea	4001	Camera	YOLOv8-RCAA	Precision=0.9823 Recall=0.8534 F1-Score=0.9133 mAP=0.9814	10.3390/agriculture14081240
Thai et al. (2025)	Banana	40114	Camera	EF-CenterNet	F1-Score=0.5688	10.1016/j.compag.2025.109927
He Y. J. et al. (2025)	Passion Fruit	6993	Camera	Transformer	Precision=0.93 Recall=0.88 F1-Score=0.9 Accuracy=0.91	10.3390/agriculture15070733
Long et al. (2025)	Tomato	3625	Online	Graph-CenterNet	Precision=0.9901 Recall=0.9276 F1-Score=0.96	10.3390/agronomy1503 0667

Current methods lack the ability to generalize across datasets, despite their strong performance on particular datasets. Building a robust universal detection framework will become an important research direction. In terms of detection timeliness, early disease identification research is in its infancy, mainly limited by the difficulty of data acquisition and the finer morphological characteristics of early lesions. Because the initial symptoms are not obvious, it is a major challenge to accurately identify the disease type and locate the focus area. However, early warning plays a key role in controlling the spread of pathogens, so it is important to develop early detection technology based on weak feature recognition. In addition, the existing detection system still relies on manual auxiliary operation.

## 4 Advances in plant leaf disease classification

The technical ability to process and analyze disease images in order to detect and differentiate between various disease categories is known as plant disease recognition technology. This technology serves as a crucial foundation for the prompt prevention and efficient treatment of plant diseases.

In the early stages of plant disease recognition research, feature extraction and screening relied heavily on artificial experience and domain knowledge. The core of its technical performance lies in whether it has two characteristics: one is whether it can fully express the essential information of disease targets and maintain high discrimination among categories. The other is whether it can form effective collaboration with classification algorithms. Traditional methods mainly use three kinds of visual features-shape, color, and texture-to classify and model. Texture features use gray level co-occurrence matrix, fractal dimension, Gabor filter, and other tools. Fractal dimension in particular is a useful tool for measuring the subtle variations in disease spot surface roughness.

Research practice shows that fusing multi-dimensional features and constructing a classifier ensemble system is the main technical path to improve accuracy. However, this artificial feature engineering poses a dual challenge to the practitioner’s professionalism: it requires both a deep accumulation of image processing technology and a knowledge reserve of plant pathology. Multiple interference elements, such as plant development stage difference, disease polymorphism, and environmental light change, make dynamic optimum adjustment of artificially built characteristic systems challenging to achieve. Traditional methods’ recognition accuracy tends to decline dramatically in complex field circumstances, particularly when the symptoms display atypical symptoms or multiple disruptions. This constraint derives mostly from the difficulties of using artificial feature modeling to adequately address the complexity and diversity of illness representations in real-world circumstances.

**Figure 5:**
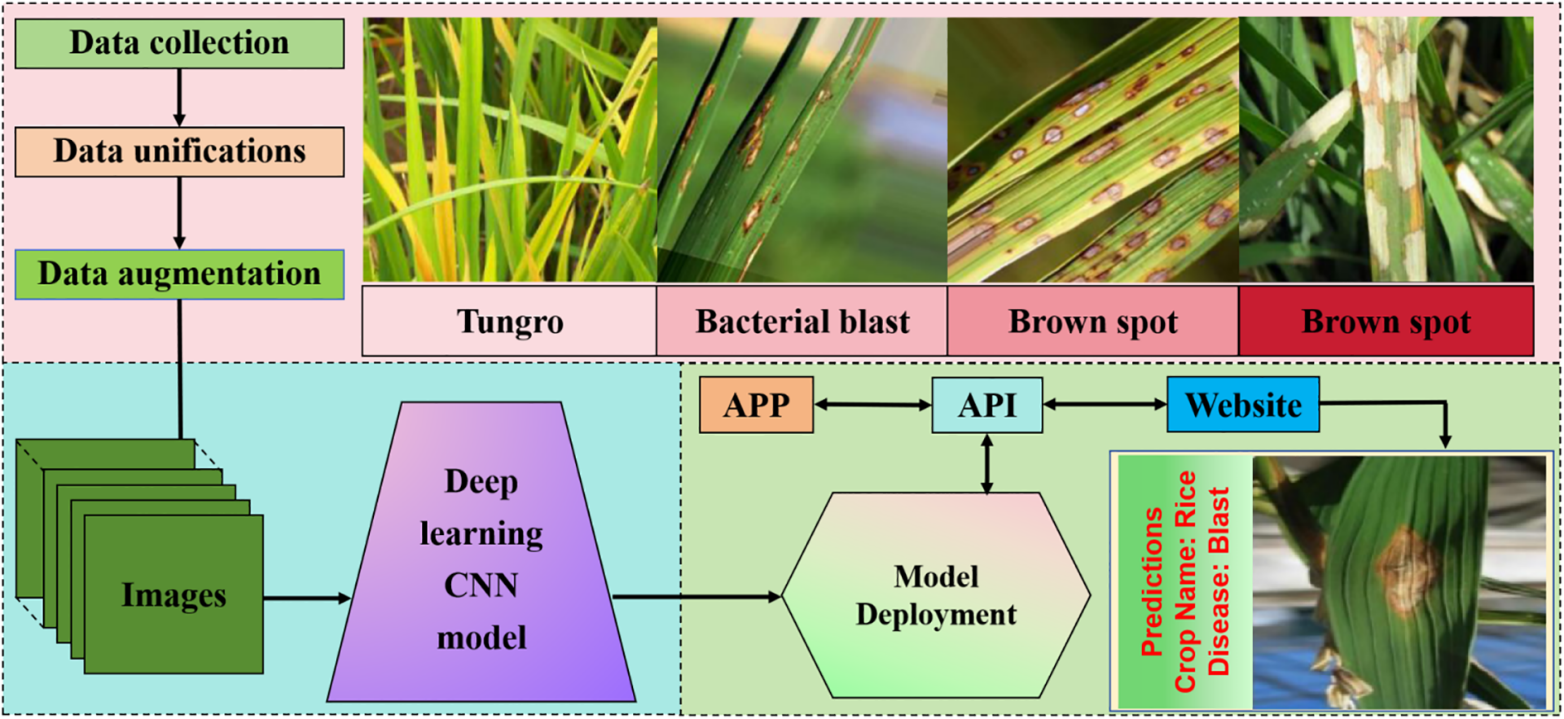
Neural network recognition based on rice leaf diseases. Image preprocessing begins by gathering image data for our project. It includes data unification and augmentation where we amalgamate multiple datasets and generate synthetic data. Our proposed deep learning model which takes an image as input and classifies it into one of the disease categories based on the features of the image. The deployment of our model which includes an application programming interface (API), an android app,and a website.

Deep learning learns feature representations directly from original image pixels through adaptive general algorithms, fundamentally breaking through the limitations of traditional artificial feature engineering. A number of significant convolutional neural network architectures have been developed as a result of changes in computing paradigms. These models include ZFNet in 2013, VGG and GoogleNet in 2014, ResNet in 2015, and later versions of DenseNet (2017), MobileNet (2017), and EfficientNet (2019). Through cutting-edge techniques like residual connection, deep separable convolution, and composite scaling, these network designs keep developing iteratively, progressively creating a technical ecosystem that can adjust to the demands of many scenarios. In general, deep learning-based techniques for identifying plant diseases adhere to the fundamental flow depicted in **Figure 5**.

### 4.1 Plant leaf disease identification based on deep neural networks

Compared with traditional plant leaf disease detection methods, the deep learning method realizes the automation of image processing flow by constructing an end-to-end recognition framework, effectively reduces manual operation links, and significantly improves detection efficiency. The independent learning ability of the model improves steadily as its complexity and network depth rise. It can record not only the global morphological aspects of leaf lesions but also multidimensional feature information, including cell structure and texture gradient. However, while the parameter scale of the deep model increases, it is also accompanied by the increase of model training time cost, gradient disappearance, overfitting, and other risk problems. Thus, by enhancing the network topology structure, adding an attention mechanism, and refining the regularization strategy, researchers are actively investigating the use of convolutional neural network architecture in the field of plant pathology detection and are consistently improving the model’s recognition accuracy for multi-category diseases and its generalization ability in complex environments.

Shah et al. (2023) introduced a pre-trained model for citrus plant disease recognition and classification utilizing the transfer learning approach and a convolutional neural network (EfficientNetB3, ResNet50, InceptionV2, and InceptionV3). In this experiment, the EfficientNetB3 model has the highest test accuracy, reaching up to 99.58%. Haleem et al. (2022) designed a robust disease detection method based on convolutional neural networks, which uses CNN’s powerful feature extraction ability to detect diseases in fruit and leaf images. The feature extraction pipeline of several of the most advanced pre-trained networks is fine-tuned to achieve the best detection performance. The average accuracy of the optimal model on the test image set is 96.6%. It has 90% accuracy. This indicates how accurate the prediction was. It works well in categorization situations where the cost of false alarms is considerable. To cut down on errors, it must now give top priority to choosing highly accurate models. Bijoy et al. (2024) demonstrated a dCNN-based model for detecting five types of rice leaf diseases: brown spot, rice blast, bacterial blight, sheath blight, and rice blast. They compared their model with 21 benchmark frameworks and 14 parallel methods and verified the effectiveness of this method through a large number of experimental results. The accuracy rate of this method reached 99.81%.

Lobna et al. (2024) used a large-scale dataset of 70,834 images to compare the performance of a tomato leaf disease classification and recognition method based on an optimized Capsule Neural Network (CapsNet) to a traditional convolutional neural network, achieving an accuracy rate of 96.39%. Kanda et al. (2022) presented a residual neural network approach for identifying tomato illnesses. The study examined variety at four levels: depth size, discrimination learning rate, training and validation data segmentation ratio, and batch size. Five network depths were utilized to determine the network’s correctness for experimental analysis. The experimental results reveal that this method outperforms prior competitive methods in tomato leaf disease identification, achieving 99.5% accuracy. Sk and Arnab (2022) demonstrated a new deep learning model based on initial layers and residual connections, which used deep separable convolution to reduce the number of model parameters. After training and testing on three plant disease datasets, the model achieved an accuracy of 99.39% on the PlantVillage dataset.

Masood et al. (2023) improved the Faster-RCNN method for computing deep keypoints and designed a deep learning method called MaizNet to locate and classify various types of maize leaf disease with an average accuracy of 97.89%, indicating the effectiveness of locating and classifying multiple types of maize leaf infection. Albahli and Masood (2022) suggested an end-to-end learning CNN structure for the efficient attention network based on the EfficientNetv2 model, which is utilized to recognize maize crop illnesses in several classes. Compared with traditional neural networks, this model has better performance, and the accuracy rate of corn crop disease classification reaches 99.89%. The framework provided by Ilyas et al. (2022) consists of three main components. The detector contained therein extracts region-based anomaly features by using a feature extractor of a deep learning network to accurately and efficiently identify and locate anomalies in plants. The algorithm achieves an average accuracy of 91.7% in anomaly detection tasks.

**Figure 6:**
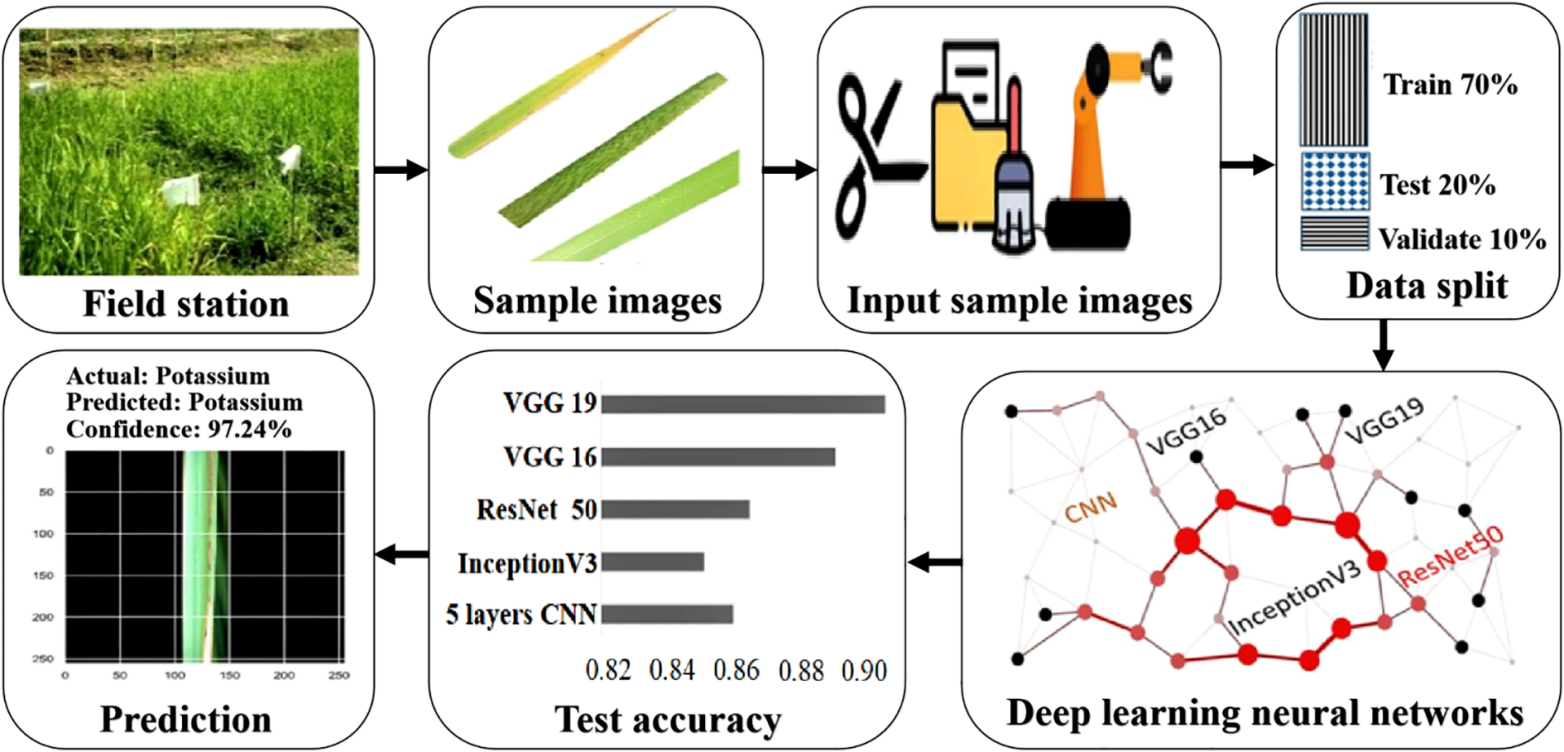
The overall rice leaf disease recognition framework. This framework is based on VGG16, VGG19, InceptionV3, ResNet50, and a 5-layer CNN to train, validate, and test open-source Kaggle plant disease data and 6 disease categories of rice leaves collected on-site in a ratio of 7:1:2.

Dey et al. (2022) proposed a deep learning-based rice leaf disease recognition framework as shown in **Figure 6** and achieved the classification accuracy of 91.8%.

Pan et al. (2024a) developed a TTN-MobileNetV2 neural network model for plant leaf disease detection based on memristor. The experimental results on the rice leaf disease dataset achieved the highest recognition accuracy of 99.16%. Pandian et al. (2022) performed a deep convolutional neural network (DCNN) model for image-based plant leaf disease recognition and trained it on an enhanced dataset of more than 240,000 images of different healthy and diseased plant leaves and backgrounds with an average classification accuracy of 98.41% on the test dataset.

Although researchers used large-scale datasets, the data’s diversity and representativeness remain insufficient. Many datasets may only include plant disease samples from specific regions, seasons, or planting settings, making it difficult to capture the complex and diverse disease circumstances seen in actual agricultural production. Plant disease performance characteristics may vary across climatic conditions, and existing datasets may not fully reflect this diversity, limiting the model’s generalizability in practical applications and making it difficult to accurately identify new environments or rare diseases.

Although researchers consistently improve model performance by strengthening network structures and integrating new mechanisms, the model’s complexity grows in tandem. Complex models not only need more computer resources and longer training times, but they may also have poor interpretability. In actual agricultural productivity, specialists are more concerned with why models produce such diagnostic results. Deep learning models behave like black boxes, making it difficult to deliver clear and understandable explanations. This has had an impact on the model’s promotion and implementation in production.

In addition, there are differences in experimental settings and evaluation criteria between different studies, which makes it difficult to make fair and objective comparisons of the performance of each model. Some studies may only be tested on specific datasets or experimental environments, without fully considering various interference factors in practical applications, such as changes in lighting, occlusion, noise, etc., resulting in a gap between experimental results and actual application effects.

Although deep learning has brought new opportunities and breakthroughs for plant leaf disease detection, in order to achieve its widespread and effective application in agricultural production, further research is needed in improving data quality, enhancing model interpretability, and standardizing experimental evaluation.

### 4.2 Plant leaf disease identification based on lightweight networks

In recent years, in order to adapt to the limitations of limited data resources in complex natural scenes, researchers have launched research on lightweight networks. Vasudevan and Karthick (2024) proposed an advanced capsule method to detect grape leaf disease. By constructing a lightweight capsule network, convolution can be separated by depth. Compared with existing deep learning models ResNeXt and traditional capsule neural networks, the computational complexity of this method is significantly reduced. Comparing the advanced Capsule network empirically with traditional CapsNet and ResNeXt models, the overall parameters and training time are much less than ResNeXt and traditional CapsNet. The accuracy of this method is 95.041%. Liu Y. et al. (2022) described a modified lightweight convolutional neural network, SqueezeNext, that included a multi-scale convolution kernel and a coordinate attention method for accurately extracting lesion information. The model achieves 91.94% recognition accuracy in the 2018 plant disease dataset. 3.02% more than the original model. The technology is suited for deployment on mobile devices and other embedded devices with modest resources, and it contributes to the popularization of intelligent agriculture. Dai M. et al. (2023) constructed an enhanced lightweight model based on GoogLeNet architecture. Compared with GoogLeNet based on Inception-V1 and Inception-V3, the model’s requirements are reduced by 52.31% and 86.69%, respectively. Compared with AlexNet, ResNet-50, and MobileNet-V2, the accuracy of this model reaches 97.87%, which is significantly higher than other models. The identification precision and calculation performance of pepper leaf diseases have advantages, which is beneficial to further large-scale popularization and application in pepper planting. The recall rate and F1-score of this method are both about 99%. Recall is suitable for recognition scenarios with high missed detection costs. At this point, it needs to prioritize ensuring a high recall rate to reduce the risk of missed detections. However, it also needs to be balanced against precision. Adjust the classification threshold based on task requirements to avoid indicator distortion caused by data distribution or threshold sensitivity. The F1-score, as a comprehensive indicator of accuracy and recall, directly reflects the model’s ability to balance “reducing misjudgments” and “avoiding missed detections”. The higher the F1 score, the more balanced the model performance, and tasks that are sensitive to both false positives and false negatives are more likely to choose high F1-score models. A lower F1-score may indicate that the model is biased towards conservatism or radicalism. It needs to adjust the threshold or optimize the model structure based on task requirements to improve overall performance.

Ma et al. (2023) explored a model that can recognize apple leaf images with complex backgrounds and disease symptoms. Model_Lite outperforms MobileNet, ShuffleNet, SqueezeNet, and GhostNet in terms of average recognition accuracy while using far fewer parameters and processing resources. The maximum grouping model’s average recognition accuracy improves by 0.19% while maintaining the same parameters and computational resources. By reducing the number of convolution kernels, the network model is reasonably simplified. The final model is a model with fewer convolution kernels, and its parameters and computational complexity are 1/344 and 1/35 of the original ResNet18 model, respectively. Although the average accuracy dropped by 0.34% on the experimental dataset, Model_Lite achieved the highest recognition accuracy of 91.21% compared to lightweight networks such as MobileNet, ShuffleNet, SqueezeNet, and GhostNet.

**Figure 7:**
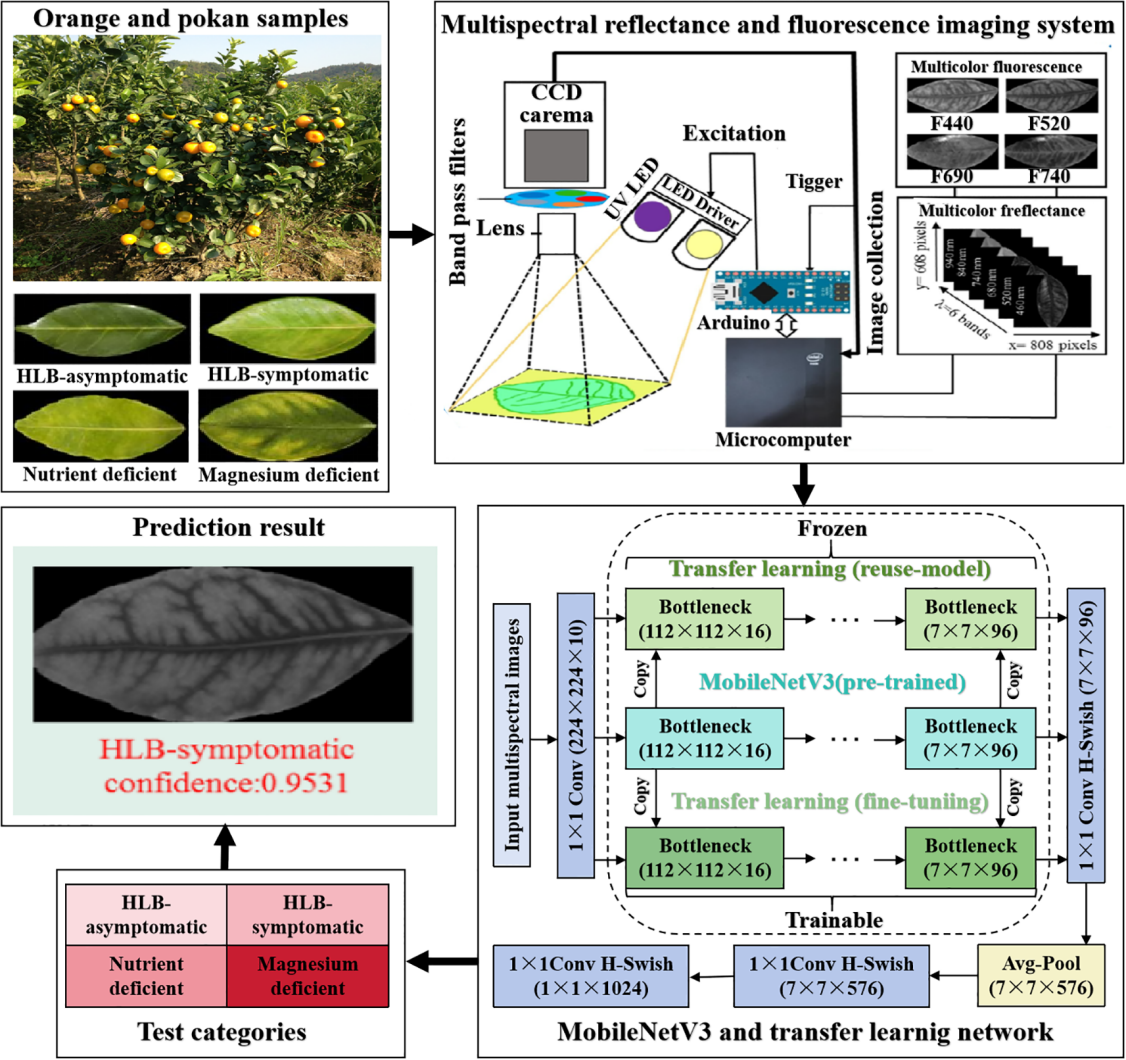
The overall orange and pokan leaf disease identification system. This system collected multispectral reflectance and fluorescence images of healthy, asymptomatic, symptomatic, magnesium-deficient, and nitrogen-deficient leaves of two varieties, Ponkan and Navel Orange, respectively. Through deep learning, the lightweight network MobileNetV3, and transfer learning, an overall classification accuracy of 92.1% was achieved.

He et al. (2022) used multi-spectral images as inputs for the MobileNetV3 and installed them on handheld edge devices to identify navel orange leaf diseases. The process is presented in **Figure 7**.

Although researchers have achieved good results in using lightweight networks for plant leaf disease recognition in complex natural scenes in recent years, there are still some problems. From the perspective of balancing model performance, some researchers have a poor grasp of the balance between accuracy and computational complexity in the pursuit of lightweighting. Although higher recognition accuracy has been achieved than MobileNet and ShuffleNet lightweight networks, the accuracy on the dataset has decreased slightly compared to the original model. This indicates that resource constraints have a certain impact on the accuracy of the model. Researchers need to further explore how to achieve more extreme lightweighting while ensuring high precision. Most existing research focuses on the detection of specific plant diseases. Although these models perform well in their respective disease detection, their universality is poor when facing complex and varied natural scenes with a mixture of multiple plant diseases. In practical applications, farmers often plant multiple crops with a wide variety of diseases. Therefore, developing lightweight plant disease detection models with broad applicability is an important challenge for the future. There are differences in the datasets, evaluation metrics, and experimental environments used in different studies, which makes it difficult to directly compare the performance of various models. Researchers should establish unified standards and norms and adopt more comparable experimental settings in order to more accurately evaluate the advantages and disadvantages of different models.

**Figure 8:**
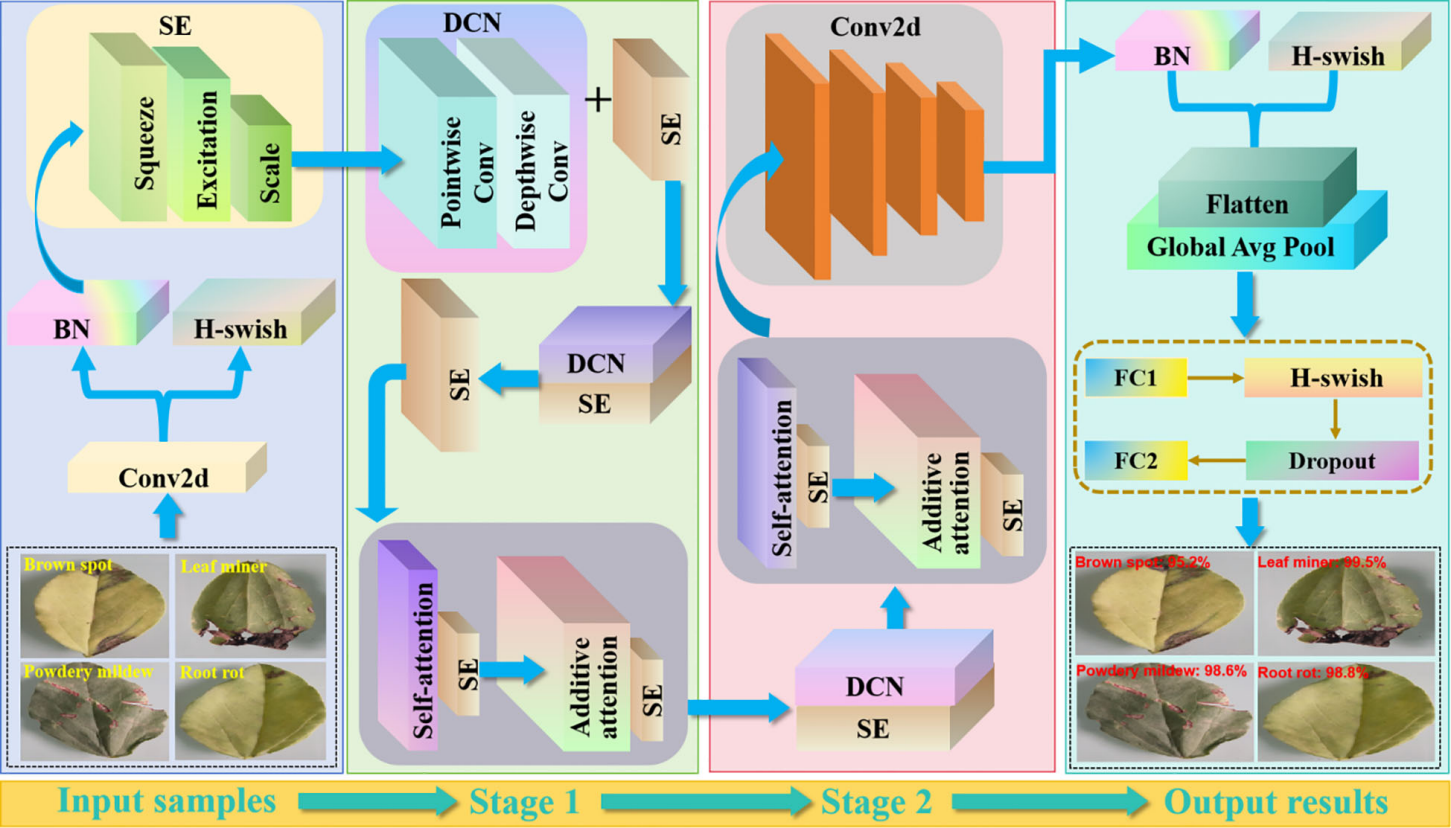
The overall pea leaf disease classification framework. MobilenetV3 adopts a lightweight network design with efficient computation and memory consumption. Compared to other networks, it has fewer parameters and can perform inference faster on mobile devices. It introduces a new Block structure and combines the squeeze and excitation (SE) module with a new activation function H-wish.

We used an improved MobileNetV3 to perform accurate pea leaf disease classification. The framework is sketched in **Figure 8**.

In summary, although lightweight networks have shown great potential in plant disease detection in complex natural scenes, further research and improvement are needed in terms of model performance balance, universality, experimental comparison, and interpretability to achieve their widespread application in intelligent agriculture.

### 4.3 Synchronous detection and recognition of plant leaf diseases

Plant leaf disease detection and identification can be done synchronously, that is, by simultaneously detecting the disease area and identifying the disease type, in addition to the methodical process of first identifying the disease and then detecting the disease area (i.e., the interested area or internet of things (IOT) area). A Siamese neural network, an effective and loosely supervised model for agricultural disease localization, was studied by Chen J. Y. et al., 2024. The model innovatively adopts a twin network structure with a weight-sharing mechanism. According to the results, ADPL-CAM performs best on all network frameworks, with an accuracy rate 27.09% higher than GradCAM and 19.63% higher than SmoothCAM. The accuracy of ADPL-CAM is 54.29%. It can accurately and timely identify and locate leaf diseases in crops. Wu et al. (2025) offered an LBPAttNet model, which incorporated the lightweight coordinate attention mechanism into ResNet18 and further extracted the local feature structure and texture features of tea diseases by using a binary mode algorithm to obtain a more comprehensive feature representation. The model achieves the highest accuracy of 98.31% in the open tea dataset, which is superior to traditional algorithms such as AlexNet, GoogLeNet, and MobileNet.

Zhang D.Y. et al. (2023) used a ResNet-50 backbone network to detect tomato leaf disease degree, outperforming the most sophisticated technique with an accuracy of 95.03%. The model is competitive in tomato disease severity and offers a novel approach to determining plant leaf disease severity. Bhatti et al. (2023) developed a new system for recognizing plant leaf diseases using Inceptionv3. They trained the model on a dataset of 80,848 photos, reaching an accuracy of 99%, and provided advice for conquering specific diseases.

Numerous creative concepts and noteworthy advancements in the identification of plant leaf diseases have been offered by researchers. They have all improved disease detection performance and accuracy to some degree, from feature extraction techniques to the development of various model architectures. But beneath these successes lies a number of unresolved issues. On the one hand, it is unclear if the model’s generalization capacity can resist the test in the intricate and constantly shifting agricultural environment, which could result in performance degradation because of environmental variations and ambiguous early disease indications. On the other hand, certain complicated models need extensive computational resources, and in real-world agricultural production settings, large-scale deployment and application are hampered by hardware constraints and costs. Furthermore, data quality and diversity challenges persist, and data collecting difficulties and annotation errors may have an impact on model training efficacy. The data improvement measures used by researchers are currently insufficiently comprehensive. To increase the practical use of plant leaf disease detection and recognition technology, constant progress in model generalization, resource consumption reduction, and data processing optimization is required.

### 4.4 Analysis and prospect of plant leaf disease identification

Convolutional neural networks’ architecture can be categorized as either deep or lightweight based on the difference in parameter magnitude. **Tables 5**, **6** systematically sort out the latest research achievements in the field of intelligent recognition of plant diseases.

**Table 5:** Plant leaf disease recognition based on deep neural network.

Authors, Years	Class	Total	Collect ways	Methods	Performance	DOI
Wang et al. (2022)	Cucumber	4740	Field shooting	Improved SwinT	Accuracy=98.97% Model Size =186.17m GFLOPs=8.78 Inference Time (ms)=71 training cost(s)=139.03	10.1016/J.COMPAG.2022.107163
Obsie et al. (2022)	Blueberry	1661	Field shooting	Yolov5s-CA	Accuracy=96.30% Precision=75.2% Recall=61.2% Model Size=13.8MB Inference Time=11.85% mAP@50 = 68.2%	10.3390/AGRICULTURE13010078
Andrew et al. (2022)	Apple	54305	PlantVillage dataset	DenseNet-121	Accuracy=99.81% F1-score=0.998 Model Size=7.05M	10.3390/agronomy12102395
Khasawneh et al. (2022)	Tomato	18160	Access via the Internet	Transfer Learning	Accuracy=99.40%	10.3390/APP 12178467
Yin et al. (2022)	Grape	4344	ImageNet dataset	GLD-DTL	Accuracy=99.84% Precision=0.995 Recall=0.995 F1-score=0.995 Model Size=30MB training cost(m)=1.15	10.25165/j.ijabe.20221503.7062
Arun et al. (2022)	Grape	240008	Access via the Internet	Conv-5 DCNN	Accuracy=98.41% Precision=0.94 Recall=1 F1-score=0.97 training cost=1000 epochs	10.3390/ELECTRONICS11081266
Yu et al. (2022)	Soybean	39446	Field shooting	RANet	Accuracy=98.49% F1-score=98.52% Precision=98.50% Recall=98.49% Model Size=42.75MB Inference Time=0.0514	10.3389/FPLS.2022.878834
Zhao Y. F. et al. (2022)	Tomato	31361	PlantVillage dataset	DoubleGAN	Accuracy=99.80%	10.1109/TCBB.2021.3056683
Prabhjot et al. (2022)	Grape	9027	PlantVillage dataset	EfficienNet B7	Precision=99.80% Accuracy=98.70% Recall=99.00% F1-score=97.00%	10.3390/S22020575
Fan et al. (2022)	Apple	3568	Apple Leaf	InceptionV3	Accuracy=98.46%	10.1016/J.COMPAG.2022.106892
Nag et al. (2023)	Tomato	18160	PlantVillage dataset	Improved CNNs	Accuracy=99.85%	10.1016/J.COMPELECENG.2023.108995
Zhang Y. K. et al. (2023)	Apple	10000	Field shooting	BCTNet	Accuracy=85.23% Recall=78.97% Model Size=79.04M training cost=300 epochs Inference Time=33FPS mAP=85.23% Map@50 = 90.65%	10.1016/J.COMPAG.2023.108132
Xu et al. (2023)	Wheat	7239	Field shooting	RFE-CNN	Accuracy=99.95% Precision=99.73% Recall=98.24% Model Size=1.235M training cost=900 epochs Inference Time=1.5s/image	10.1016/J.PMPP.2022.101940
Saidani et al. (2023)	Rice	20000	PlantVillage dataset	Based on PlantNet	Accuracy=97% Model Size=2.47M Inference Time=0.04s	10.31577/cai_2023_6_1378
Daniya and Vigneshwari (2023)	Rice	6213	Rice disease dataset	RWW-NN	Accuracy=90.7%	10.1016/J.ADVEN-GSOFT.2023.103472
Cui and Tian (2023)	Rice	2500	Field shooting	YOLO v3	Accuracy=91.84% Precision=91.12% Recall=91.84% F1-score=91.87% training cost=1500 epochs mAP=86.72%	10.3390/AGRICULTURE13010170
Buchke and Mayuri, 2024	Tomato	21000	PlantVillage dataset	EfficientNett	Accuracy=99.50% Precision=0.9950 Recall=0.9950 F1-score=0.9950	10.21203/rs.3.rs-3149045/v1
Zhang R. F. et al. (2023)	Tomato	16453	PDDA, PlantVillage	IBSA_Net	Accuracy=99.70% Precision=0.989 Recall=0.993 F1-score=0.991	10.3390/APP13074348
Hu B. et al. (2023)	Apple	54303	PlantVillage dataset	FOTCA	Accuracy=99.80% F1-score=0.9931 Model Size=59.14M training cost=11 epochs	10.3389/FPLS.2023.1231903
Liang and Jiang (2023)	Tomato	22930	PlantVillage dataset	ResNet 50-DPA	Accuracy=99.28% Precision=99.29% Recall=99.28% F1-score=99.28% training cost=200 epochs	10.3389/FPLS.2023. 1258658
Liu et al. (2023)	Apple	3171	PlantVillage dataset	Inception-V3	Accuracy=99.45% Precision=99.84% Recall=99.10% F1-score=99.00%	10.1109/TCBB.2022.3195 291.
Md et al. (2023)	Rice	3710	Rice Leaf dataset	PlantDet	Accuracy=98.53% Precision=98.50% Recall=98.35% F1-score=98.42%	10.1109/ACCESS.2023.3264835
Cui et al. (2023)	Tree	259800	Field shot	DINO-ViT	Accuracy=96.95%	10.3390/PLANTS12183280
Wang B. B. et al. (2023)	Apple	54306	PlantVillage dataset	ULEN	Accuracy=98.13% Precision=98.13% Recall=97.49% F1-score=97.76% Model Size=111758P Inference Time=0.037s	10.1007/s11119-023-10020-0
Liu and Zhang (2022)	Grape	2056	Field shooting	GLDCNet	Accuracy=99.57% Precision=98.48% Recall=98.49% F1-score=98.99%	10.1016/J.COMPAG.2024.108668
Wang H. M. et al. (2024)	Maize	3686	Field Shot	TC-MRSN	Accuracy=99.59% Precision=94.88% Recall=93.21% F1-score=93.52% Model Size=5M	10.1016/J.COMPAG.2024.108915
Hatice and Veysel (2024)	Potato	1500	Muhammad	MDSCIRnet	Accuracy=99.33%	10.1016/j.engappai.2024.108307
Farian and Neema (2024)	Grape	4040	Access via the Internet	CNN+RF	Accuracy=95.34% Recall=99.00% F1-score=99.00%	10.1016/J.HELIYON.2024.E33377
Qiu et al. (2024)	Tomato	18363	Access via the Internet	Improved AlexNet	Accuracy=98.83% Precision=99.77% Recall=99.15% F1-score=99.40%	10.1016/J.HELIYON.2024.E33555
Kasana and Rathore, 2024	Potato	7101	PlantVillage dataset	XceptionNet	Accuracy=97.25%	10.3390/APP 14178038
Zhang E. X. et al. (2024)	Tomato	50000	PlantVillage dataset	YOLO v4	Accuracy=98.17% Precision=98.73% Recall=98.69% F1-score=98.71% Model Size=0.91M	10.3389/FPLS.2024.1420584
Pan et al. (2024b)	Rice	60235	PlantVillage dataset	Mobile Net V2	Accuracy=99.16% Model Size=5.79MB	10.1109/ACCESS.2024.3444796
Hicham et al. (2024)	Bean	3296	Field shooting	YOLO-NASM	Accuracy=88.80%	10.3233/JIFS-23615 4
Rashid et al. (2025)	Tomato	18835	PlantVillage dataset	MobileNetV3	Accuracy=98.77% Precision=99.00% Recall=99.00% F1-score=99.00% training cost=4h	10.1109/ACCESS.2025.3550205
Zhu et al. (2025a)	Citrus	2283	CCL’2	YOLOv5	Accuracy=96.1% Precision=90.4% Recall=90.90% Model Size=14.3MB Inference Time=61.77FPS mAP@0.5 = 92.1%	10.3390/sym17040617
Islam et al. (2025)	Rice	30000	Online	PlantCareNet	Accuracy=97% Precision=97% Recall=97% F1-score=97% Model Size=19.2MB Inference Time=0.0021s/	10.1186/s13007-025-01366-9
Kumar and Bidarakundi (2025)	Coffee	58555	Arabica	Coffee-Net	Accuracy=99.95% Precision=97.36% Recall=97.25% F1-score=96.88%	10.1109/ACCESS.2025.3525661
Tang et al. (2025)	Maize	5796	PlantVillage dataset	ResNet50	Accuracy=98.79% Precision=88.63% Recall=88.33% F1-score=88.28%	10.1109/ACCESS.2025.3525661
Gaashani et al. (2025)	Maize	4188	Online	MSCPNet	Accuracy=97.44% Precision=96.76% Recall=97.37% F1-score=97.04% Inference Time=0.0111s	10.1109/ACCESS.2024.3524729
Raza et al. (2025)	Potato	3261	Online	DENSE-NET-121	Accuracy=99.08% Precision=98.00% Recall=96.00% F1-score=97.00%	10.1016/j.heliyon.2025.e42318

**Table 6:** Plant leaf disease recognition based on lightweight network.

Authors, Years	Class	Total	Collect ways	Methods	Performer	DOI
Liu S. et al. (2023)	Apple	700	Field shooting	Improved MobileNetV2	Accuracy=0.96 Precision=0.97 recall=0.92 F1-score=0.93	10.1590/fst.104322.
Sabbir et al. (2022)	Aplle	54309	PlantVillage	MobileNetV 2	Accuracy=0.99	10.1109/ACCESS.2022.3187203.
Lu et al. (2022)	Maize	21967	Internet	ECA-ShuffleNetV2	Accuracy=0.96 Precision=0.94 recall=0.95 F1-score=0.94	10.3390/AGRICULT- URE12111929.
Samia et al. (2023)	Apple	61486	PlantVillage	MULTINET	Accuracy=0.63 Precision=0.65 F1-score=0.58	10.1109/ACCESS.2023.330 3868.
Kang et al. (2023)	Maize	2775	Field shooting	CenterNet	Accuracy=0.85 Precision=0.91 recall=0.61 F1-score=0.69 mAP=0.85	10.3390/APP131810441.
Dai G. W. et al. (2023)	Leaf	4503	open data sets	PPLC-Net	Accuracy=0.99	10.1016/J.JKSUCI.2023.101555.
Zhang R. et al. (2024)	Rice	3357	Field shooting	YOLO-CRD	Accuracy=0.90	10.32604/PHYTON.2024.052397.
Cheng et al. (2024)	Rice	1500	Field shots	D-R-C-YOLOv7-Tiny	Accuracy=0.92 Precision=0.92 recall=0.82 F1-score=0.87 mAP=0.92	10.3390/AGRICULTURE1405 0709.
Shwetha et al. (2024)	Jasmine	2000	Field shooting	LeafSpotNet	Accuracy=0.97 Precision=0.94	10.1016/J.AIIA.2024.02.002.
He and Tong, 2025	Tomato	3641	Online	LT-YOLO	Accuracy=0.90	10.326 04/cmc.2025.060550.
Sun et al. (2025)	Tomato	800	Camera	Faster RCNN	Accuracy=0.97 recall=0.85	10.3389/fpls.2024.1491593.

The existing database of plant disease images (both proprietary and open-source) exhibits a notable spatial distribution divergence. In contrast to the variable scenes faced by agricultural plant protection robots in field operations (e.g., light fluctuation, branch occlusion, and dynamic shooting angle), the current research paradigm primarily focuses on the controlled environment of human-computer cooperation (such as standardized shooting distance and fixed viewing angle), as evidenced by the disease target area, which is primarily concentrated in the center of the image and accounts for more than 60% of the image. Systematic experimental analysis reveals that the recognition accuracy of convolutional neural networks optimized on specific datasets (e.g., ResNet-50 with 98.2% accuracy in the PlantVillage) can drop by 12%-15% when migrated to cross-domain disease image libraries, emphasizing the importance of adaptive model architecture screening for different application scenarios. Although some studies have investigated mobile disease identification technologies (e.g., lightweight deployment based on TensorFlow Lite), their reasoning delays are typically greater than 200 milliseconds (EfficientNet-B0 takes approximately 340 milliseconds per frame under the MXNet framework), making it difficult to meet the millisecond response requirements of real-time plant protection decisions.

The study’s content and network model selection are changing dramatically in the deep learning application to plant disease recognition. In terms of research content, the focus has shifted from improving the disease recognition accuracy of convolutional neural networks to optimizing the operation efficiency. In addition, the research scene has also shifted from a single background in the laboratory to disease recognition in a complex background in the natural environment, and the detection method has also expanded from static image analysis to dynamic video surveillance. At the same time, the scope of disease feature extraction has been expanded from single-leaf disease to multiple plant organs, including root, stem, leaf, flower, and fruit. In terms of network models, researchers are moving from traditional networks such as VGG, GoogleNet, ResNet, and DenseNet to lighter network structures such as MobileNet and EfficientNet. This shift aims to reduce model parameters and speed up operations without sacrificing model performance to meet the needs of artificial intelligence (AI) edge computing platforms with limited computing resources.

## 5 Synthesis

When using deep learning for plant pathology research in various agricultural settings, it is vital to select technical solutions flexibly based on individual circumstances and needs, as well as optimize practical benefits by merging cutting-edge technology. Because of available computational resources and stringent recognition accuracy criteria, the two-stage detection model should be preferred for high-precision analysis scenarios in the laboratory. High recognition accuracy can be achieved on typical datasets by divorcing the architecture of the regional recommendation network from the classification branch. However, it must accept the trade-off between high model complexity and poor inference speed, making it more appropriate as a core tool for disease mechanism research and precise diagnosis. Real-time monitoring scenarios in the field require a compromise between detection speed and dynamic environmental adaptability. The majority of detectors are either one-stage (YOLOv8, SSD optimized version) or anchor-free (CenterNet). The former considerably improves inference efficiency by implementing an end-to-end prediction method. The latter employs keypoint estimate rather than anchor box generation, ensuring great robustness in occlusion and complex backdrops. They can all support the real-time processing requirements of field mobile devices. Deep separable convolution and channel pruning approaches can be utilized to compress model parameters to less than 5 million, adjusting to the computational limits of low-power devices.In addition, the weakly supervised localization recognition method achieves lesion localization through image-level labels, further reducing annotation costs and providing a feasible solution for model deployment in resource-limited areas.

Future technological integration can concentrate on three key areas: To begin, we introduce the Transformer self-attention mechanism, which dynamically captures the long-range dependencies of disease regions, increasing model detection stability in scenarios with overlapping leaves and uneven lighting. Second, provide semi-automatic annotation tools that prioritize annotating high-value samples using self-learning methodologies, lowering annotation costs by more than 60% while maintaining data quality. Third, using neural architecture search (NAS) technology, heterogeneous network architectures are automatically generated for difficult field situations such as multi-scale illness spots and background interference. A lightweight architecture that merges dilated convolution and attention modules via reinforcement learning search achieves both accuracy and efficiency optimization. The following methodologies can help to improve the application of deep learning models in agricultural contexts, driving the progress of plant pathology technology toward precision and universality.

The actual field setting presents numerous obstacles, including reciprocal blockage of leaves, changes in sunlight, and complicated backdrops such as dirt and weeds. The available approaches are generally weakly resilient. When there is severe occlusion or an exceedingly complex backdrop, the performance of both the first-stage and second-stage detectors may suffer greatly. The anchor-free box approach uses center points or keypoints to provide relatively consistent performance under occlusion. Because of the lack of exact spatial information learning, lightweight recognition networks and weakly supervised algorithms are especially susceptible to background interference. The adaptation to occlusion improves the model’s robustness in complicated, changing field situations.

In terms of annotation cost, fully supervised object identification algorithms rely on a huge amount of precisely annotated bounding boxes and category information, and the annotation cost is quite high, making the technology difficult to implement. Lightweight recognition networks just need image-level labels like “disease” or “health,” as well as disease categories, which considerably reduces the annotation burden and makes them more feasible for large-scale classification systems. In scenarios when annotation resources are severely restricted, weakly supervised localization recognition techniques offer an alternate solution by utilizing image-level labels to find lesions. However, their localization accuracy and robustness are typically lower than fully supervised systems.

At the model deployment level, although researchers have successfully deployed models on edge devices, there are significant differences in computing power, storage capacity, and power consumption among different edge devices. This poses a great challenge to the adaptation of the model. Some models that perform well on high-performance devices may not be able to run at all or run at extremely slow speeds on resource-limited devices, which cannot meet the needs of real-time detection. For example, deploying complex deep learning models on low-end agricultural sensor devices with extremely limited resources still faces technical challenges. The maintenance and update mechanism after model deployment is also incomplete. The agricultural production environment is constantly changing, and new diseases continue to emerge. The characteristics of the original disease may also change. If the model cannot be updated in a timely manner, its detection accuracy will gradually decrease. However, model updates often require the collection of large amounts of data and retraining of the model, which is costly, time-consuming, and difficult to implement quickly on agricultural sites.

Performance trade-off is an unavoidable issue for deep learning models in agricultural disease detection. Accuracy, speed, and computational resource consumption are the three key indicators for measuring model performance. In practical applications, there is often a mutually restrictive relationship between them. Some models adopt complex network structures and a large number of parameters to improve detection accuracy. Although this enables the model to achieve high accuracy on specific datasets, it also brings problems of excessive computational resource consumption and slow detection speed. In agricultural fields, especially in large-scale farmland, it is necessary to quickly detect diseases in a large number of crops. Slow models cannot meet the actual production pace, which may lead to delayed detection and treatment of diseases, resulting in serious economic losses. On the contrary, in order to pursue detection speed and reduce computational resource consumption, some lightweight models have been proposed. However, these models often have insufficient accuracy and may result in false positives or false negatives. For example, in complex backgrounds or situations where early symptoms of diseases are not obvious, lightweight models may not be able to accurately identify diseases, which can affect disease prevention and control. How to find the optimal balance between accuracy, speed, and computational resource consumption is one of the urgent problems that deep learning needs to solve in agricultural disease detection.

The transferability of models to different regions is another major challenge for the application of deep learning in agricultural disease detection. There are differences in climate, soil, and planting variety factors in different regions. These differences can lead to variations in the types, patterns, and symptoms of plant diseases. Currently, most deep learning is trained and validated on datasets specific to a particular region. When these models are applied to other regions, their performance often deteriorates significantly due to differences in data distribution. For example, when the rice disease detection model trained in the southern region is applied to the northern region, it may not accurately identify the unique rice diseases in the north. In addition, the level of agricultural management and planting patterns in different regions can also affect the applicability of the model. Some regions may adopt advanced agricultural technologies and management methods. The crop growth environment is relatively stable, and the occurrence of diseases is relatively regular. Other regions may have outdated agricultural technology, complex and variable planting environments, and unpredictable disease occurrence. This makes the application of the same model in different regions require a lot of adjustment and optimization according to the local specific conditions, increasing the difficulty and cost of model application.

In conclusion, while deep learning has promising applications in agricultural disease detection, there are still many issues with model deployment, performance trade-offs, and model transferability between areas. Only through ongoing research and innovation to address these critical concerns will deep learning technology actually play a vital role in agricultural production and global food security.

## 6 Conclusions

With the help of deep learning technology, plant disease target detection and recognition studies have become a popular topic in scientific research circles both at home and abroad. A substantial body of research has greatly increased the accuracy of plant disease identification and contributed significantly to technological progress in this field. However, in actual implementations, these deep learning-based solutions encounter numerous challenges, and there is still a long way to go before fully and efficiently solving the problem of plant disease detection and diagnosis.

Data acquisition challenges: creating high-quality data takes a significant amount of time, labor, and money. Images of diverse plant varieties, disease types, stages of plant diseases, and other conditions should be gathered during the data set construction phase. In the processing step that follows, individuals with specialized knowledge should correctly identify the status of the plant disease.

Data imbalance: There is less data on plant diseases that are difficult to collect and rare than on plant diseases that are easy to collect and common, resulting in insufficient learning of deep learning models and affecting plant disease recognition accuracy.

Data noise interference: During the acquisition process, the image may be affected by external factors such as uneven natural lighting, a complex plant background, a poor shooting angle, and shooting height. These factors will interfere with the extraction and learning of plant disease features.

Computational resource requirements: Deep learning models have complex structures, and training and prediction require powerful computational resources. This increases the research threshold and research cost of plant disease identification.

Model optimization is difficult: Deep learning models have overfitting and underfitting problems, and hyperparameter adjustment requires a lot of experiments and rich experience. Problems such as vanishing or exploding gradients may also occur during model training, affecting the convergence and performance of the model.

Poor model interpretability: Because deep learning models are a black box, it is difficult to intuitively understand the models’ base and procedure for judging plant illnesses, as well as to troubleshoot and optimize.

Environmental adaptability: the field environment is complex and changeable, and the conditions, such as light, temperature, and humidity, are greatly different. The model performs well in the laboratory environment, but its performance may decrease in actual field application.

High real-time needs: large-scale agricultural output, the necessity for real-time disease detection, and the model’s reasoning speed all place increased demands on the model; some complicated models struggle to satisfy real-time requirements.

High deployment and maintenance costs: To deploy the model to the actual production environment, it is necessary to consider the compatibility of hardware equipment, network communication, data security, and other issues. Later model updates and maintenance also require professional technicians and resources.

Plant disease identification enters a promising era of change. In the field of deep learning, new models and algorithms are constantly emerging, such as transformers, lightweight neural networks, etc. With its excellent feature extraction ability, the deep learning method can capture the subtle features of plant diseases, thus significantly improving the accuracy and reliability of recognition.

With the advancement of sensor technology, plant physiological and environmental factors can be obtained more conveniently and comprehensively in the process of data collection so as to obtain higher quality plant disease data sets. In addition, multi-source data fusion creates conditions for building more accurate disease identification models. Plant leaf disease identification, a crucial component of precision agriculture, has drawn attention from a variety of sources, been supported by national resources, and accelerated the field’s growth as a result of the acceleration of the agricultural modernization process and the growing demand for precision agriculture.

Deep learning-based plant leaf disease detection has also seen significant prospects as interdisciplinary collaboration has grown. Combining computer science, agronomy, biology, and other fields allows us to examine plant disease occurrence settings, disease development characteristics, transmission rules, and identification techniques from a variety of perspectives. Because computer science has strong algorithms and data processing capabilities, it offers effective technical support for disease detection. From the standpoint of plant development environment and cultivation management, agronomy offers valuable practical expertise and important hints. Biology reveals the pathogenic mechanism of pathogens and the defense mechanism of plants by examining biological disorders at the microscopic level. The technique for identifying plant diseases will be advanced from various perspectives thanks to this multidisciplinary study. We have good reason to think that accurate early warning, comprehensive real-time monitoring, and effective plant disease prevention and management will soon be achieved, safeguarding both sustainable agricultural development and global food security.

## Glossary

IEEE: the institute of electrical and electronics engineers
CVPR: computer vision and pattern recognition
IDADP: agricultural disease and pest research database
PDDB: plant disease symptom database
AdaBoost: adaptive Boosting
SVM: support vector machine
R-CNN: region-based Convolutional Neural Networks
YOLO: you only look once
SSD: single shot multi-box detector
PWD: pine wilt disease
RPN: region proposal network
RoIAlign: region of interest align
RoIPool: region of Interest Pooling
CBAM: convolutional block attention module
CNN: convolutional neural network
VGG: visual geometry group
ECA: efficient channel attention
IoU: intersection over union
SE: squeeze and excitation
mAP@0.5: mean average precision at IoU=0.5
API: application programming interface
CapsNet: capsule neural network
DCNN: deep convolutional neural network
IOT: internet of things
AI: artificial intelligence
NAS: neural architecture search.
